# The atomic structure of the Bergman-type icosahedral quasicrystal based on the Ammann–Kramer–Neri tiling

**DOI:** 10.1107/S2053273319017339

**Published:** 2020-02-11

**Authors:** Ireneusz Buganski, Janusz Wolny, Hiroyuki Takakura

**Affiliations:** a Faculty of Physics and Applied Computer Science, AGH University of Science and Technology, Al. Mickiewicza 30, Krakow, 30-059, Poland; b Graduate School of Engineering, Hokkaido University, Sapporo, Hokkaido 060-8628, Japan; c Faculty of Engineering, Hokkaido University, Sapporo, Hokkaido 060-8628, Japan

**Keywords:** Bergman quasicrystal, atomic structure, average unit cell

## Abstract

In this study, the atomic structure of the ternary icosahedral ZnMgTm quasicrystal (QC) is investigated by means of single-crystal X-ray diffraction. The structure is found to be a member of the Bergman QC family, frequently found in Zn–Mg–rare-earth systems. The *ab initio* structure solution was obtained by the use of the *Superflip* software. The infinite structure model was founded on the atomic decoration of two golden rhombohedra, with an edge length of 21.7 Å, constituting the Ammann–Kramer–Neri tiling. The refined structure converged well with the experimental diffraction diagram, with the crystallographic *R* factor equal to 9.8%. The Bergman clusters were found to be bonded by four possible linkages. Only two linkages, *b* and *c*, are detected in approximant crystals and are employed to model the icosahedral QCs in the cluster approach known for the CdYb Tsai-type QC. Additional short *b* and *a* linkages are found in this study. Short interatomic distances are not generated by those linkages due to the systematic absence of atoms and the formation of split atomic positions. The presence of four linkages allows the structure to be pictured as a complete covering by rhombic triacontahedral clusters and consequently there is no need to define the interstitial part of the structure (*i.e.* that outside the cluster). The 6D embedding of the solved structure is discussed for the final verification of the model.

## Abstract 2

The article discusses the atomic structure modelling based on the Ammann–Kramer–Neri tiling of the ternary Bergman quasicrystal in the 3D real space.

## 1. Introduction   

### 1.1. General information   

Among complex metallic alloys the atomic structure of a quasicrystal (QC) is without a doubt the most difficult to solve. Attempts at structure solution of icosahedral quasicrystals (iQCs) date back to the early 1980s, and not long after the publication of the paper by Shechtman *et al.* (1984 ▸) the first model of an AlMn alloy was proposed (Guyot & Audier, 1985 ▸). Even though almost 35 years have passed, there exists no unified approach to conducting the structure determination, in contrast to periodic crystals. Over the years, the higher-dimensional approach has become the most successful way to handle aperiodic structures with long-range order (Katz & Duneau, 1986 ▸; Kalugin *et al.*, 1985 ▸; Elser, 1985 ▸; Bak, 1985 ▸). The desired and well-known periodicity is restored by lifting the atomic structure to the *n*-dimensional (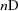) space, where *n* > 3. That mathematical construction allows one to calculate a structure factor in a concise, simple way. Without it, the comparison between the structure model and the experimental diffraction data could not be done (Yamamoto, 1996 ▸).

Even though the periodicity is re-established in 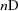 space, the structure solution is still a complex task. The atom in 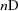 space is no longer a point object but an object with a bulk geometry called the occupation domain (OD) (*e.g.* Yamamoto & Takakura, 2004 ▸; Steurer, 1990 ▸) that is extended in the internal (also called phasonic, perpendicular) space. The internal space represents all additional dimensions that are necessary to restore translational symmetry in higher dimensions. The real-space coordinates of the QC are found as an intersection of the 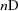 crystal with a 3D real (physical, parallel) space. The main problem of the 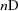 approach is that the detailed shape of the OD is unknown. The structure solution of QCs in 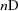 relies on the determination of the OD’s shape and the distribution of elements within.

The iQCs, besides decagonal QCs, are the most frequently observed QCs in intermetallic compounds (Steurer, 2018 ▸). They are in fact the only QCs that are aperiodic in every direction of the 3D space, at least taking into account crystals displaying the ‘forbidden’ crystallographic symmetry. So far, only the face-centred icosahedral (FCI) and primitive icosahedral (PI) phases have been reported experimentally. The only observation of the body-centred icosahedral phase (I-type) comes from numerical simulations (Engel *et al.*, 2015 ▸; Subramanian *et al.*, 2018 ▸). iQCs are divided into three groups depending on the local geometry of atomic sites. There are Mackay QCs frequently found in Al-based systems (Audier & Guyot, 1986 ▸; Mackay, 1962 ▸), Bergman QCs formed in Zn-based QCs (Bergman *et al.*, 1952 ▸; Henley & Elser, 1986 ▸) and the latest to be discovered, Tsai QCs, found in Cd/CaYb alloys (Tsai *et al.*, 2000 ▸; Guo *et al.*, 2000 ▸). In spite of the above-mentioned examples there are numerous exceptions, *e.g.* ZnSc is a Tsai-type phase (Yamada *et al.*, 2016 ▸), whereas AlCuLi (Dubost *et al.*, 1986 ▸) is a Bergman phase. When it comes to the stabilization mechanism iQCs are considered to be Hume-Rothery phases (Hume-Rothery, 1926 ▸), where the number of valence electrons per atom in the structure is *e*/*a* ≃ 1.8 for Mackay QCs, ≃ 2.1 for Bergman QCs and ≃ 2.15 for Tsai QCs (Tsai, 1998 ▸).

### 1.2. The icosahedral setting and the Ammann–Kramer–Neri tiling   

An X-ray diffraction experiment is the primary source of structural information in crystallography. The diffraction pattern of the iQC can be indexed with six vectors that form a 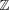-module. There are two frequently used settings of those vectors: Cahn’s (Cahn *et al.*, 1986 ▸) and Elser’s (Elser, 1986 ▸). The latter was chosen in this research as the software that was used for the structure solution and refinement utilizes this setting. Each vector of the reciprocal space is a sum of the base vectors 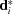: 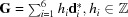, where
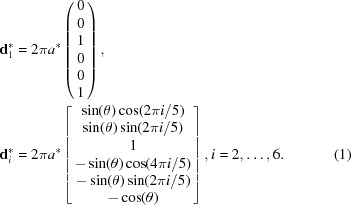



**Figure 1:**
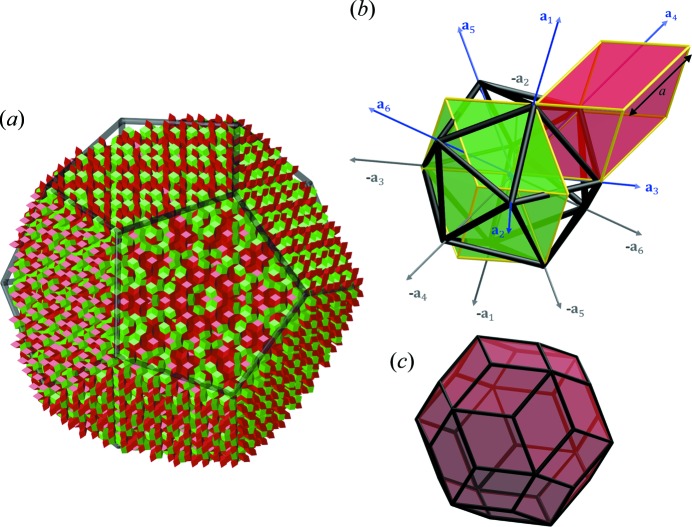
(*a*) The 3D representation of the AKNt generated by the cut-and-project method. Two types of rhombohedra are plotted in different colours. (*b*) The definition of the icosahedral setting in a 3D real space. Vectors are directed from the centre of the icosahedron towards six vertices. The AR (red) and the OR (green) are shown to be spanned by vectors of the icosahedral setting. (*c*) The rhombic triacontahedron that defines the shape of the OD for the AKNt.

The angle 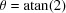. The vectors of the direct space can be obtained from the orthogonality condition 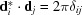:
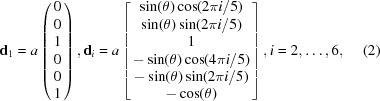
where 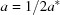. The 6D lattice constant 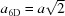. The first three coordinates, either for the 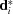 or the 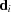, are the set in the real space and the last three coordinates are the set in the internal space, so 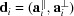, 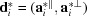. That will be particularly important when the procedure of lifting the 3D structure to 6D is discussed in Section 7. For simplicity, the real-space components of the 6D basic vectors will be further called 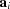. Vectors 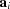 are directed from the centre towards the vertices of the icosahedron [Fig. 1 ▸(*b*)].

The structure of the iQC is generally interpreted using the Ammann–Kramer–Neri tiling (AKNt) (Kramer & Neri, 1984 ▸), popularly called 3D Penrose tiling [Fig. 1 ▸(*a*)]. We prefer to call it AKNt to pay tribute to the original researchers who discovered the tiling. The AKNt is not used directly in the 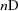 approach but the subset of its 12-fold vertices, namely 12-fold sphere packing vertices, defines the centres of atomic clusters. The truncated triacontahedron, which represents the distribution of the 12-fold vertices in a 6D space (Henley, 1986 ▸), is used to construct the ODs. In a real space, the AKNt is built out of two rhombohedra: prolate (AR), also called acute, and oblate (OR), also called obtuse [Fig. 1 ▸(*b*)]. Those two golden rhombohedra can cover a whole 3D real space without gaps or overlaps between adjacent rhombohedra, resulting in the icosahedral 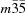 point-group symmetry. The norm *a* of the vectors 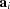 is equal to the edge length of the rhombohedra. In contrast to the Penrose tiling, the matching rules for the AKNt are unknown, as are the inflation/deflation rules for rhombohedra, apart from weak matching rules (Hann *et al.*, 2016 ▸). Recently, a method to construct an icosahedral packing was described by Madison (2015 ▸), but four golden zonohedra, instead of just two golden rhombohedra, are used in this framework. The AKNt can be generated from 6D space by the cut-and-project method. The projection is executed by the use of the projection matrix 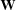 whose *i*th column is equal to the vector 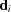. The OD has in this case the shape of the rhombic triacontahedron [Fig. 1 ▸(*c*)] positioned in the vertices of the 6D unit cell. If the OD is placed in the centre of the unit cell the dual AKNt is obtained (Mihalkovič *et al.*, 1996 ▸).

### 1.3. Review of the Bergman iQC and motivation   

The interest in icosahedral Frank–Kasper phases (FK) (Frank & Kasper, 1958 ▸) increased with the discovery of the FCI AlMgLi (Niikura *et al.*, 1993 ▸) and AlMgPd (Koshikawa *et al.*, 1992 ▸). Their structure is built out of the Bergman cluster which is formed from the dense packing of tetrahedra. After the report by Luo *et al.* (1993 ▸) that the iQC is formed in ZnMgY many iQC phases were found in ZnMgRE (RE, rare-earth element) ternary alloys. The first to be grown were FCI phases with Y, Tb, Dy, Ho and Er (Niikura *et al.*, 1994*a* ▸). After a series of investigations, PI phases were also grown by rapid solidification with Nd, Sm, Gd, Tb, Ho, Er, Tm, Yb, Lu and Y (Niikura *et al.*, 1994*b* ▸). Apart from the iQC, the decagonal QC is also found in ZnMg*M* (*M* = Y, Dy, Ho, Er, Tm and Lu) (Sato *et al.*, 1998 ▸). The atomic diameter was shown to play a role in the stabilization of the decagonal phase. A similar mechanism was observed for ZnMg-based alloys with a third, trivalent element (Kaneko *et al.*, 2001 ▸). The larger the atomic radius, the higher the concentration of the trivalent element in the QC. What is more, for atomic radius ∼1.65 Å the PI phase is preferred, rather than the FCI phase, which is formed for atomic radius ∼1.75 Å.

The atomic structures of both PI and FCI Bergman QCs were studied in the AlCuLi (Yamamoto, 1992 ▸) and AlMgLi (Tsai *et al.*, 1994 ▸) systems, respectively. The structure of AlCuLi was first investigated by Elswijk *et al.* (1988 ▸) constituting a so-called EHSB model based on the simple decoration scheme (Elser & Henley, 1985 ▸). The simple decoration scheme assumes the atomic decoration of two original rhombohedra in vertices and mid-edges. The AR is additionally decorated with two atoms on a longer body-diagonal, dividing it in a proportion τ/1/τ, where τ is the golden ratio. The next take on the structure was made by Yamamoto who introduced major modifications to the EHSB model (Yamamoto, 1992 ▸) by removing mid-edge atoms from the AR and replacing them with edge-off-centre positions. Additionally, the positions of the 12-fold vertices were left unoccupied as they created a short distance with edge-off-centre atoms. The decoration was settled to reflect the local atomic arrangement in the *R*-AlCuLi cubic approximant as the strong affinity of the QC and periodic approximant was known. The same decoration was proposed to explain the structure of cubic AlZnMg (Henley & Elser, 1986 ▸). Based on the local information derived from the approximant crystal, the 6D model of the QC could be constructed. The model is not reliable due to strong mixing of Al, Cu, Li atoms. Later, the model of the FCI AlMgLi was proposed (Tsai *et al.*, 1994 ▸). The same decoration as for the PI AlCuLi was used, but the reconstruction of the superlattice reflections required two icosahedral lattices, with even- and odd-parity nodes.

The more recent model of the Bergman QC was proposed for the PI ZnMgHo QC (Takakura & Yamamoto, 2007 ▸). The model was based on the result of the *ab initio* phasing of the diffraction amplitudes with LDEM (low-density elimination method) (Takakura *et al.*, 2001 ▸, 2006 ▸), implemented in *lodemac* (Takakura *et al.*, 2001 ▸) and known structures of the FK approximants (Kreiner, 2002 ▸; Sugiyama *et al.*, 2002 ▸). The model was founded on the same framework as the model of the CdYb iQC (Takakura *et al.*, 2007 ▸). Instead of Tsai, Bergman clusters are assumed to occupy the subset of the 12-fold vertices of the AKNt. The additional shell of the rhombic triacontahedron (RTH), which encompasses the so-called ‘glueing’ atoms, is considered among traditional shells of the Bergman cluster. The same cluster expansion was originally proposed for Tsai-type QC approximants. A whole model was set in a 6D space. The three ODs, vertex-centred (OD_V_), body-centred (OD_B_) and edge-centred (OD_E_), are created from the archetype truncated triacontahedron generating the 12-fold vertex environment of the AKNt. The choice of such an archetype OD ensures that only two types of links between clusters are possible: the *b* linkage along a twofold direction and the *c* linkage along a threefold direction. The *a* linkage along a fivefold direction is excluded as it is not observed in approximant crystals and is said to create short interatomic distances. Unfortunately, the model by Takakura *et al.* of the Bergman QC does not include the interstitial atoms, which are atoms outside the cluster. In the Tsai-type QC the space between clusters is filled with OR and AR decorated according to the simple decoration scheme observed in 1/1 and 2/1 cubic approximants (Gómez & Lidin, 2001 ▸, 2003 ▸). In the Bergman QC, a unique decoration of two rhombohedra does not exist. It is a local-environment dependent; therefore the complete shape of the OD is difficult to obtain.

The present work on the structure of the Bergman QC was motivated by two major factors. Firstly, the Zn–Mg–RE QCs are still investigated with respect to magnetic properties and the search for long-range magnetic order (Goldman, 2014 ▸). The RE ions provide the well-localized magnetic moments originating from the 4*f* electrons. The problem of moment interaction in the quasilattice could therefore be resolved. So far only the spin-glass-like behaviour at low temperature has been observed with no magnetic long-range order. The existing model of the RE atom distribution in the FCI Bergman phase does not explain the moment’s frozen state (Sato, 2005 ▸).

Secondly, our research is driven by the deep desire to acquire a reliable model of the Bergman QC, which is especially relevant now since the recent discovery of superconductivity in AlZnMg iQCs (Kamiya *et al.*, 2018 ▸). That discovery fascinated not only researchers in the area of aperiodic crystals but also the community working in strongly correlated electron systems. This is an ideal time to extend the potential application of the QCs and explore their peculiar properties. Knowledge about the structure of the Bergman QC will play a major role in this venture.

The article is organized as follows. Section 2 presents the details of the experiment. In Section 3 the *ab initio* structure solution based on the result of the charge-flipping algorithm is presented. The methodology, which helps to build the initial model for the structure refinement, is explained in Section 4. Section 5 is dedicated to the presentation of the result of the structure refinement. The RTH cluster covering is discussed in Section 6, followed by Section 7 dedicated to the 6D embedding of the refined structure. In Section 8 the result of the structure refinement is discussed with attention paid to the RE atom distribution and the disorder.

## 2. Experimental details   

Single grains of the ZnMgTm iQC were grown by a solution growth technique (Canfield & Fisk, 1992 ▸). High-purity elements Zn (Nilaco, 99.99%), Mg (Nilaco, 99.99%) and Tm (Rare Metallic Co. Ltd, 99.9%) with the nominal composition of Zn_62.8_Mg_33.6_Tm_3.6_ were put in an alumina crucible and sealed inside a silica ampoule together with 0.333 atm (1 atm = 101.325 kPa) of argon. The elements in the crucible were melted at 1023 K for 5 h and cooled to 863 K using a muffle furnace with a cooling rate of 2 K h^−1^. After holding at this temperature for about 50 h, the silica ampoule was quenched in water. The grains of the Zn–Mg–Tm iQC, which have a shiny fracture surface, were embedded in a solidified solution. The chemical composition of the iQC was determined to be Zn_69.5_Mg_20.9_Tm_9.6_ by wavelength-dispersive X-ray spectroscopy (WDX) on an electron probe microanalyser (EPMA: Jeol, JXA-8530F).

The single-crystal X-ray intensity data were collected on a single-crystal diffractometer (Rigaku XtaLAB PRO MM007) with Mo *K*α (λ = 0.71073 Å) radiation at ambient temperature. A specimen of size 0.07 × 0.06 × 0.04 mm was cut from a large single grain for use in the experiment. The intensity data set was processed using the software package *CryAlis PRO* (Rigaku Oxford Diffraction, 2015 ▸). A total of 1 190 808 reflections were observed, which were reduced to a data set of 3953 unique reflections, assuming the space group 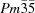, and indexed with sextet integers with *R*_int_ = 0.062. Of these 3010 reflections met the condition for *F*_o_ > 3σ(*F*_o_). The icosahedral lattice constant *a* was determined to be equal to 5.128 (3) Å.

## 3. The *ab initio* structure solution   

The derivation of the initial model for the refinement of aperiodic crystals, especially QCs lacking the average, periodic structure, has become much more convenient since the algorithm for the phase retrieval of diffraction amplitudes was invented. There are two iterative algorithms serving the purpose: LDEM (Takakura *et al.*, 2001 ▸) implemented in the *QUASI07_08* package (Yamamoto, 2008 ▸) and the charge-flipping algorithm (Oszlányi & Sütő, 2004 ▸) used in the *Superflip* software (Palatinus, 2004 ▸). The *ab initio* phase retrieval allows one to obtain a value of the crystallographic *R* factor of around 14–18% in most cases, which helps immensely to construct an atomic model of the structure. The phase retrieval could not be so accurate without the availability of high-quality diffraction data. Fortunately, modern detectors allow the collection of hundreds of thousands of diffraction peaks with a high precision.

The 3010 symmetrically independent reflections were used in a phase retrieval and subsequently used to calculate the electron density. The *ab initio* structure solution with *Superflip* resulted in *R* = 14.5% which allows us to consider that the electron density calculated with the retrieved phases of the diffraction peaks approximates well a real structure. At this stage the preliminary analysis of the structure began.

**Figure 2:**
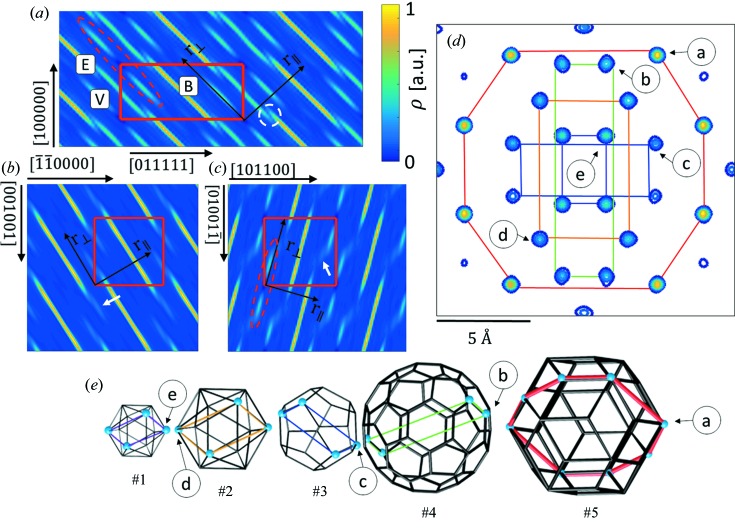
The 2D high-symmetry sections through the 6D electron density, obtained by the phase retrieval, containing (*a*) fivefold, (*b*) twofold and (*c*) threefold axes both in parallel space (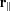) and perpendicular space (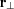); (*d*) the section through the RTH cluster centre showing the pattern characteristic of the Bergman cluster. All the five shells are shown in (*e*) as a 3D object. The plane section through each shell is marked with the same colour in (*d*) and (*e*) for guidance.

The standard practice is to calculate the 2D high-symmetry sections through the electron density in the 6D space. From the sections, general information on the atomic distribution in the structure can be obtained. In Fig. 2 ▸(*a*) the section containing two perpendicular fivefold axes [100000] and [011111] is presented. Three ODs are manifested: OD_V_ (marked V), OD_B_ (marked B) and OD_E_ (marked E). Those three ODs are known from the previous study of ZnMgHo (Takakura & Yamamoto, 2007 ▸), which is isostructural to ZnMgTm. In contrast to the Tsai-type iQC, where cluster centres are generated by the OD_B_, in this structure the OD_V_, enclosed in the red dashed ellipsoid, generates the positions of the 12-fold vertices of the AKNt. The empty electron density, manifested in the centre of the OD_V_, means the cluster centre sites should be unoccupied. In addition, the electron density in the OD_V_ is relatively small, meaning RE elements do not occupy those positions. Tm atoms are expected to occupy the positions generated by the OD_B_, as a high concentration of the electron density is located there. Furthermore, in the same figure the white dashed circle shows the overlay of the OD_E_ and the OD_B_. That is the possible phason flip site that occurs along a fivefold direction in the 3D real space.

In Fig. 2 ▸(*d*) the section through the cluster centre in the electron density in 3D real space is shown. The image is perpendicular to a twofold axis. The characteristic pattern formed by all the five shells of the Bergman cluster is presented. The shells are the following: small icosahedron, large icosahedron, dodecahedron, soccer-ball polyhedron and the RTH. The classical Bergman cluster shells are expanded by considering the RTH shell as a part of the cluster. Each of the high-electron-density sites in Fig. 2 ▸(*d*) is related to its corresponding place in Fig. 2 ▸(*e*) showing the 3D shape of each shell. The highest concentration of the electron density occurs at the nodes of the #5 shell. It means that the RE elements occupy all the vertices of the last shell of the RTH cluster.

In Fig. 2 ▸(*d*) no splitting of atomic sites can be perceived but in sections (*a*)–(*c*) the markers indicating such a splitting occurs are detected. In Figs. 2 ▸(*b*) and 2 ▸(*c*) the white arrow shows the splitting of the electron density along a twofold and a threefold direction, respectively, along the perpendicular space component of the 6D space. Additionally, the electron density is shifted along a parallel space direction. That means, in the atomic model, that split atomic positions are highly probable. Furthermore, the splitting of the atoms along a twofold direction occurs perpendicularly to a radial direction in the cluster as a radial direction is always directed along a threefold and a fivefold axis.

**Figure 3:**
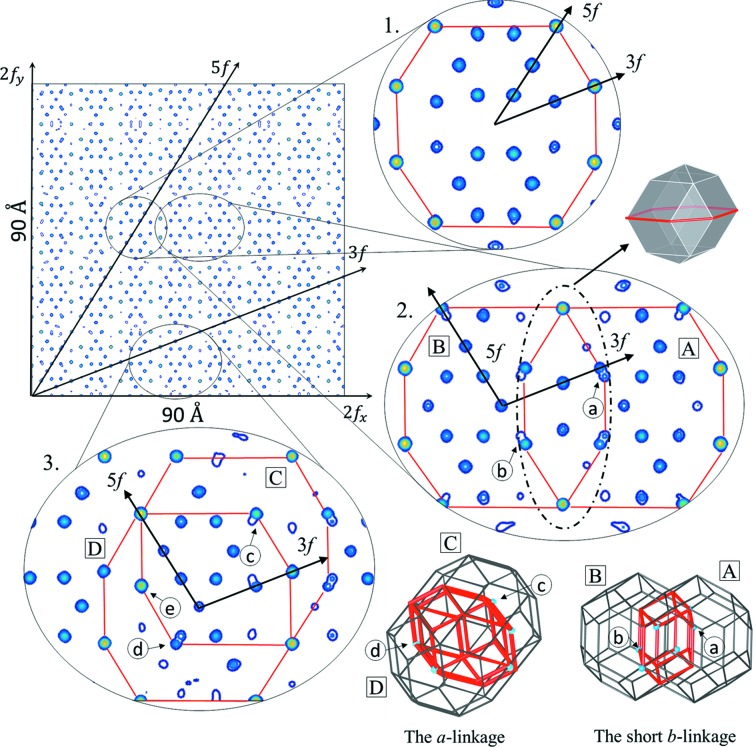
The 2D section through the 3D electron density. The area of 90 × 90 Å calculated in the plane perpendicular to a twofold direction is shown. Three patches are highlighted: (1) the undistorted Bergman cluster; (2) the short *b* linkage; (3) the *a* linkage. The 3D visualization of both linkages is also presented.

In order to further explore the *ab initio* structure solution, an extended 2D section through the 3D electron density is presented (Fig. 3 ▸). An area of 90 × 90 Å was calculated perpendicularly to one of the twofold axes. Such an orientation of the section allows us to find the location of clusters easily. Three patches of the electron density were selected and magnified for further discussion.

The top patch (1) in Fig. 3 ▸ shows a very clear, undistorted electron-density distribution. The high concentration of the density occurs at the sites corresponding to the nodes of the #5 shell of the RTH cluster. Those are the Tm atom positions. Inner shells are also occupied by a rather heavy element like Zn or mixed Zn/Tm atoms. The cluster centre in this case is empty, confirming the result of the ZnMgHo study (Takakura & Yamamoto, 2007 ▸).

Patch (2) shows two clusters, ‘A’ and ‘B’, linked together by the short *b* linkage with a length of 8.9 Å. The intersection of two RTH clusters along the short *b* linkage forms a rhombic dodecahedron with small pyramids on the top and bottom, represented in Fig. 3 ▸ both in the form of a 2D section and a 3D visualization. Such a linkage is not observed in the approximant crystals and is not part of the 12-fold sphere packing set; therefore the traditional model that constitutes the cluster approach that was used for the Tsai-type CdYb iQC cannot incorporate such a linkage. Interestingly, the cluster centre in both clusters is not empty and this site is occupied by a rather heavy atom. It is however not the feature of the short *b* linkage, but the *a* linkage formed between those two presented clusters that is shown in patch (3). The presence of the central atom caused a deformation of the inner sites generated by the small icosahedron (#1 shell) along a fivefold direction. Site ‘a’ of the cluster ‘A’ is generated by the OD_E_, which was shown to intersect with the OD_B_ in Fig. 2 ▸(*a*) along a fivefold direction, leading to the phason flip. The site ‘a’ is shared with the neighbouring cluster ‘B’. This site in the cluster ‘B’ is part of the #5 shell which is generated by the OD_B_. That is exactly the phason flip site from Fig. 2 ▸(*a*) marked with a white dashed circle. The same reasoning applies to the site ‘b’.

In the last patch (3), the *a* linkage along a fivefold direction is shown. The intersection of two RTH clusters forms a rhombic icosahedron. The formation of the *a* linkage is visualized in 3D. The *a* linkage appears in the 12-fold vertices of the AKNt, but according to the standard practice it is removed from the cluster model as it creates too short interatomic distances and has not been seen in approximant crystals. Indeed, the positions, which are created by the #5 shell of the cluster ‘D’ and by the #1 shell of the cluster ‘C’, are very close to each other but do not overlie [position ‘c’ (‘d’)]. Consequently, distortion of the electron density along a fivefold direction is created. What is interesting is that once again the centres of the clusters connected via the *a* linkage are occupied by a rather heavy atom. This atom can be interpreted both as a central atom and the atom occupying the node of the #2 icosahedral shell of the neighbour cluster. The other interesting position is the site ‘e’ occupied by a heavy atom. It can be interpreted as the position generated by the node of the triacontahedron ‘C’ or the node belonging to the #3 dodecahedral shell from the cluster ‘D’. That means that the Tm element can be found either on the #5 triacontahedral shell or on the #3 dodecahedral shell.

**Figure 4:**
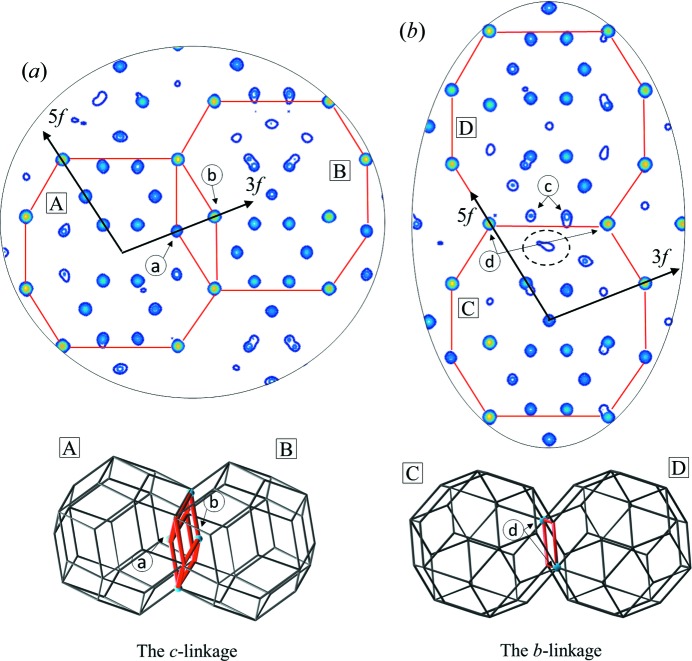
(*a*) The section through the *c* linkage occurring in the structure of icosahedral ZnMgTm with RTH clusters marked with a red line. The 3D visualization is provided. Atoms ‘a’ (‘b’) are shared by the #5 shell of cluster ‘B’ (‘A’) and the #3 shell of cluster ‘A’ (‘B’). (*b*) The section through the *b* linkage occurring in the structure. The split atomic sites ‘c’ are indicated.

A separate figure is dedicated to the *b* and *c* linkages (Fig. 4 ▸). Due to the existence of the *a* linkage Tm elements were said to occupy the #3 dodecahedral shell. However, it is not the only reason. As pointed out in Fig. 4 ▸(*a*) the *c* linkage (12.2 Å) also contributes to sharing atoms from the #5 triacontahedral shell with the #3 dodecahedral shell. Atoms ‘a’ and ‘b’ are mutually exchanged between two clusters forming the *c* linkage. In contrast to the *a* linkage the positions overlie ideally, and no atomic splitting is generated. Nevertheless, it can be concluded that Tm atoms occupy both the #3 shell and #5 shell as a by-product of the *c* and *a* linkages.

Interestingly, the *b* linkage (14.1 Å) also causes a small amount of atomic disorder in the structure. The atomic sites ‘c’ in Fig. 4 ▸(*b*) are part of the #4 shell of the cluster ‘D’. The same atoms should appear in the cluster ‘C’. However, due to the short distance, they are absent. What remains is a weak electron density enclosed with a dotted line. We can expect the atomic sites ‘c’ to form a split atomic position as well. Positions ‘d’ are shared by clusters, but no disorder is created.

**Figure 5:**
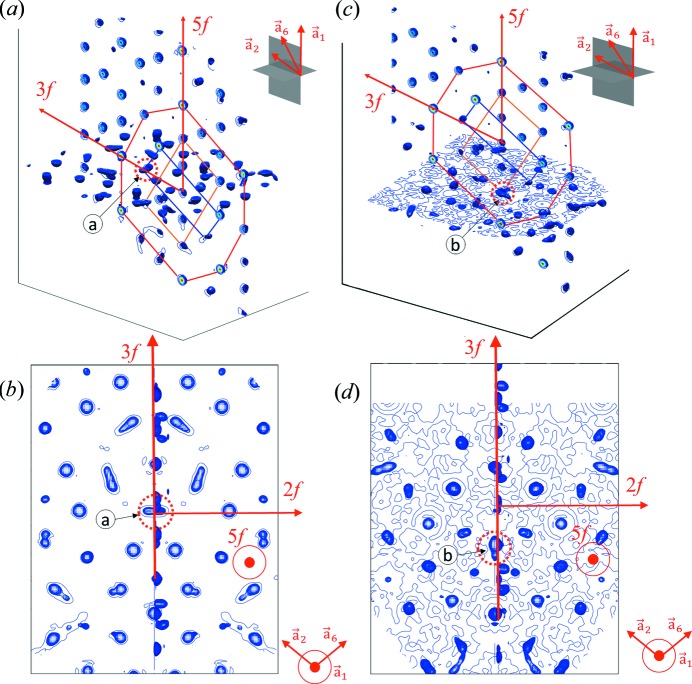
The isosurface plot in two perpendicular planes with the contour slice plot (in the vertical plane) for the 3D electron-density map. (*a*) The isometric view with the position ‘a’ indicated by an arrow and dotted circle and recognized as the split atomic site on the dodecahedron (section through the dodecahedron marked with dark blue line). The splitting occurs perpendicularly to the cluster’s radial direction which is a twofold direction and corresponds to the position indicated by the white arrow in Fig. 2 ▸(*b*). The outline of the RTH cluster is drawn with the red line. Additionally, the outline of the outer icosahedron is marked with an orange line; (*b*) the top view of (*a*). (*c*) The isometric view with the position ‘b’ recognized as the split atomic site on the outer icosahedron along a threefold direction. Such a split position corresponds to that indicated by the white arrow in Fig. 2 ▸(*c*). The contour of the RTH cluster is drawn with the red line alongside the contour of the #2 icosahedron that is marked with an orange line and the contour of the #3 dodecahedral shell marked with a dark blue line; (*d*) the top view of (*c*). The specific directions are indicated with red arrows. The positions of the planes in which the electron densities are calculated in the isometric views in (*a*) and (*c*) are pictured with grey planes together with the orientation of the vectors in the icosahedral setting.

In order to show the splitting of the atomic sites along a threefold and a twofold direction, the isosurfaces and a contour plot were calculated (Fig. 5 ▸). In Fig. 5 ▸(*a*) the splitting of one of the atoms belonging to the #3 dodecahedral shell is shown. The site ‘a’ is located on a threefold axis directed from the centre of the cluster, and is shown in the picture (red line). Fig. 5 ▸(*b*) presents the top view of the contour plot, along a fivefold axis. The splitting is shown to occur along a twofold axis, perpendicularly to the radial direction of the cluster. Since the OD_B_ generates the positions on the nodes of the dodecahedron, this splitting corresponds to the situation indicated by the white arrow in Fig. 2 ▸(*b*). Fig. 5 ▸(*c*) shows the splitting of the atomic site ‘b’, belonging to the #2 icosahedral shell. In Fig. 5 ▸(*d*) the splitting is shown to occur along a threefold axis. This is the splitting indicated by the white arrow in Fig. 2 ▸(*c*), occurring for the OD_V_, which generates the positions on the #2 icosahedral shell.

## 4. The structure model   

The infinite structure model of the Bergman ZnMgTm iQC was constructed by ascribing the atomic decoration to two golden rhombohedra in the AKNt. Even though the AKNt is indirectly used in all the models of iQCs, *i.e.* it provides the 12-fold vertex environment and defines the atomic decoration of the interstitial part, it has never been utilized as a quasilattice for which the prototiles are building blocks of the structure. The main reason why it has never been used is that the unique decoration of rhombohedra cannot be determined in real structures (Janssen *et al.*, 2018 ▸; Qiu *et al.*, 1996 ▸; Qiu & Jarić, 1995 ▸). To our knowledge, the latter statement has never been tested, nor proven for the inflated tiling. PI QCs obey the 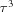 scaling rule (Ogawa, 1985 ▸). Therefore, the edge length of the golden rhombohedra can be inflated from a regular 5.13 Å to 21.7 Å. We faithfully decided to try this approach and carry out the structure modelling. Of course, we worked with a strong assumption that the structure is well modelled by the decorated AKNt, which is 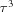 inflated.

**Figure 6:**
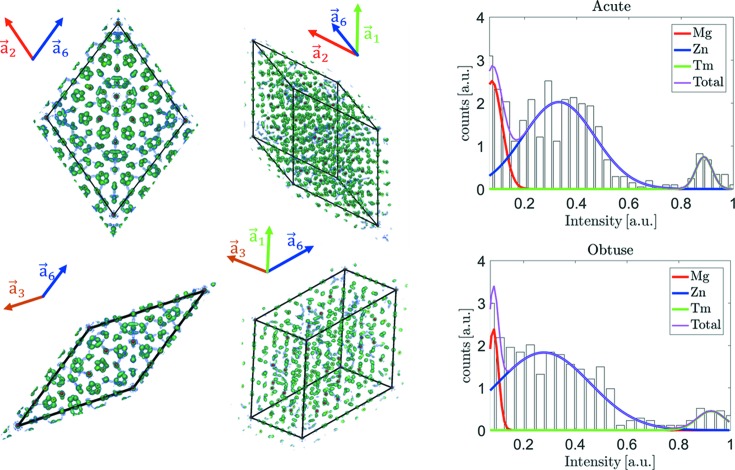
The visualization of the isosurface electron density inside the prototiles of the AKNt. The green (red) isosurface was plotted for 1/10 (1/3) of the maximal intensity. Two orientations for each rhombohedron are shown. The orientation is expressed by arrows directed towards base vectors of the icosahedral setting. The method of finding the atomic sites based on the electron density is shown. The algorithm finds the maxima which are assumed to be the centres of atoms for the initial model of the iQC. The atomic elements are ascribed to each site based on the distribution intensity in this site. Three Gaussian distributions correspond to three elements found in the sample of the ZnMgTm alloy. The strongest intensity is ascribed to Tm, whereas the weakest is Mg.

To find the decoration of the inflated rhombohedra, the electron density in randomly selected rhombohedra of the AKNt was calculated (Fig. 6 ▸). The electron density is subjected to small discrepancies between selected rhombohedra due to the limited amount of diffraction data and the phase retrieval, which although very satisfying, cannot be considered complete. The complete reconstruction would be possible only with the infinite number of diffraction peaks and that is impossible for obvious reasons. In order to minimize the electron-density variations, the average electron density was calculated over chosen rhombohedra. That helped us to obtain a good approximation of the atomic distribution inside rhombohedral units. It must be stated that the decoration of each rhombohedron was repeatable. Therefore, the 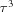-scaled rhombohedra can be considered as proper building blocks of the structure. The maxima in the electron density correspond to the real-space atomic positions. Approximately 800 positions were determined for AR and 500 for OR. Such a large number of atoms in the building blocks of the structure would be impossible to refine as only 3010 diffraction reflections are at our disposal. The number of parameters was reduced by using the symmetry of each rhombohedron. By applying the threefold rotation and mirror symmetries the volume of the rhombohedron, which is considered independent in the course of the structural refinement, was reduced by a factor of six. For the final model 148 atomic positions in AR and 104 for OR were used.

The last information required to finish the preparation of the initial model is the element distribution in the rhombohedra. Fortunately, Zn, Mg and Tm differ significantly in terms of the atomic scattering factor for X-rays; therefore they can be rather clearly distinguished in the electron-density map. In Fig. 6 ▸ we have plotted histograms of the calculated electron density for the found maxima. For both AR and OR three Gaussian-like distributions can be found. Each one corresponds to the specific element. The first Gaussian, closest to the origin, counts instances of Mg, which is the lightest element with atomic number *Z* = 12. The next broad distribution is assigned to Zn with *Z* = 30. It is also the largest distribution as the content of Zn in the iQC is around 69%. The smallest distribution belongs to Tm, which is also the heaviest element in the structure with *Z* = 69. The histogram is normalized. Three Gaussian functions were fitted to the histogram plot. The type of atom is assigned to each site by the condition of the intensity in this position. If its intensity lies within 2σ from the corresponding Gaussian function’s maximum the element is ascribed unambiguously and is not subjected to further refinement. If the intensity lies between distributions corresponding to particular elements, this position is left as a mixed site and the ratio of occupation is refined. That procedure led to the initial composition of Zn_68_Mg_24_Tm_8_, which is in good agreement with the experiment.

The crystallographic *R* factor of the starting model is equal to 35%. That value, even though large for inorganic crystals, does not negate our model. Firstly, at this stage of the investigation, the model was not refined. Secondly, the phason disorder is known to be a dominant factor affecting the intensity of the diffraction peak. Lack of phasonic correction can lead to *R* = 27% even though the atomic model of the QC is very good (Buganski *et al.*, 2019 ▸).

## 5. The structure refinement   

The structure solution and refinement were based on the real-space modelling of atomic positions. The atomic elements were assigned to specific positions in the rhombohedra and later, by assembling rhombohedral units to form the AKNt, a whole structure in physical space was recreated. The real-space structure refinement, based on the average unit cell (AUC) approach, which is known to work exclusively in the physical space, was previously used for decagonal structures (Kuczera *et al.*, 2011 ▸, 2012 ▸). Its main principle is the construction of the atomic distribution function (Wolny, 1998 ▸; Wolny *et al.*, 2018 ▸; Buczek & Wolny, 2006 ▸). The distribution arises from projection of all positions on the periodic, reference lattice. In practical applications, only the reference vertex of the rhombohedra must be projected because positions of atoms are related to that chosen site by vector translation. The AUC approach has never been used for the structure refinement of iQCs; therefore, a whole methodology including the code for the structure refinement had to be developed from scratch.

The refinement of the structure was conducted with the use of our own code written in the *Matlab* software environment. The library *fmincon* was used to optimize the parameter of the structure with the interior-point algorithm as a solver. The chosen solver satisfies bounds at each iteration and does not have to operate on full matrices which saves time, especially for problems with a large number of parameters. The structure-factor calculation is based on the tiling-and-decoration scheme, where the geometric part of the structure factor is separated from the atomic part (Strzalka *et al.*, 2015 ▸). The geometric part is the Fourier transform of the distribution related to one vertex of each rhombohedra, which was selected as a reference site. The atomic positions are given exactly with respect to the coordinates of the reference site. The geometric component is multiplied by the sum of the plane waves scattered over each atom, multiplied by the phononic Debye–Waller factor and the atomic scattering factor.

In the structure refinement, 763 free parameters in total were refined. Those include: atomic coordinates, phononic atomic displacement parameters (ADPs) (isotropic), phasonic ADPs in a general Debye–Waller formula (Bancel, 1989 ▸; Lubensky *et al.*, 1986 ▸) (one parameter for a whole structure), extinction parameter (Coppens & Hamilton, 1970 ▸), and a scale factor between experimental and calculated structure amplitudes. Additionally, for the mixed atoms the partial occupation probability for each element was refined, with the restriction that all have to sum to 1. Since many atoms of the model are placed on the edges or the faces of the reduced units of rhombohedra, the coordinate positions of those atoms are fixed and are not subjected to the action of the optimization algorithm. Parameters are refined against 3010 diffraction peaks which makes the reflections-to-parameters ratio ∼3.9. The given ratio, although low for an inorganic structure, allows us to perform the structure refinement. The number of parameters is quite large due to the large size of the rhombohedra. The reduction of the size of the rhombohedra is not possible since the model is restrained by the 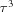 scaling and a regular size of rhombohedra cannot be decorated uniquely. The larger size (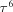-inflated one) would be irrational from the numerical point of view, since the volume of the rhombohedra would grow 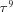 times with respect to already inflated tiles and the number of atoms would grow to tens of thousands.

**Figure 7:**
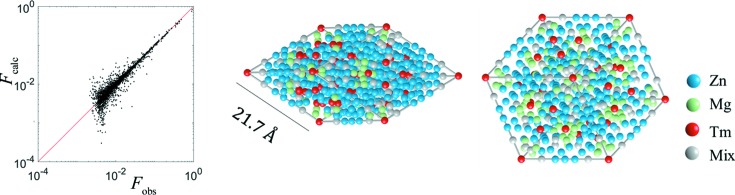
The correlation plot *F*_calc_ versus *F*_obs_ (left). *R* = 9.8%. The atomic decoration of two rhombohedra of AKNt after the refinement (right). The edge length is equal to 21.7 Å. Tm atoms are located in the vertices of rhomboidal faces and form a rhombus in the middle of each face. Those are related by the 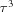 scaling property. Only 1/6th of each rhombohedron is refined.

After the structure refinement, which resulted in a very good *R* = 9.8%, no short atomic distances are observed, except for the high-symmetry sites with partially occupied atomic sites (Fig. 7 ▸). We have allowed for only a small shift of atoms from the original position (<1 Å in each cycle of the refinement program) which restrains the atoms from freely sliding within the structure. We can observe a few instances of Zn/Mg and Zn/Tm mixed occupancies, and very few where all three elements are found to be mixed (although they are mostly occupied by Zn/Mg and trace amounts of Tm). Such mixing is not unusual in Bergman phases and is observed for approximant crystals in ternary ZnMg-based alloys (Gómez *et al.*, 2008 ▸). The final composition after the refinement was concluded to be Zn_66.7_Mg_25.1_Tm_8.2_ with *e*/*a* = 2.08 and a point density equal to 0.062 Å^−3^. It is in acceptable agreement with the experiment. Nevertheless, there is a significant deviation from the experimental composition, especially considering the Zn-to-Mg ratio, which does not affect the *e*/*a* ratio. Such a deviation is rather common not only for ZnMg-based QCs but also for their approximants. The discrepancy up to 3 at.% is not rare. The question is whether a significant disorder observed in those structures, both positional and chemical in nature, is an intrinsic component that should be accepted (Henley, 1991 ▸).

## 6. The RTH covering   

The structure was refined within the scheme of the atomic decoration of golden rhombohedra forming the AKNt. The standard method of modelling the iQC and approximants is the cluster-based approach where the local atomic arrangement is expressed by three known so far families of clusters. It is interesting to see how our refined structure corresponds to the cluster model.

It is intriguing that all the atoms in our model correspond to some sites of the Bergman cluster. In fact, by covering the inflated rhombohedra with RTH clusters, we can assign every single atom to a RTH cluster forming a kind of quasiperiodic covering. A covering is usually discussed for decagonal QCs, *e.g.* the Gummelt cluster has such a property (Gummelt, 1996 ▸), but it was never elaborated nor implied for an iQC. That feat is not possible for the 12-fold sphere packing based model of iQCs. If *b* and *c* linkages are allowed exclusively, the interstitial part of the structure always arises. In our case such a distinction between RTH cluster sites and interstitial structure is unnecessary. It would be even inappropriate since there is no condition that would allow us to select the clusters that are considered ‘proper’. It is caused by the existence of the *a* linkage along a fivefold direction and the short *b* linkage along a twofold direction. Those linkages are excluded from the cluster-based approach as they create too short interatomic distances, as shown in Section 3, and are never seen in the approximant crystals. The length of each linkage, appearing in our model, is the following. The length *b* of the *b* linkage is equal to 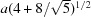 = 14.1 Å. The length *c* of the *c* linkage is equal to 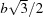 = 12.2 Å. The length of the *a* linkage is equal to the edge length of the original (not inflated) rhombohedra, that is 5.13 Å. The short *b* linkage can be calculated from the *b* linkage by subtracting the original lattice constant, resulting in 8.9 Å.

**Figure 8:**
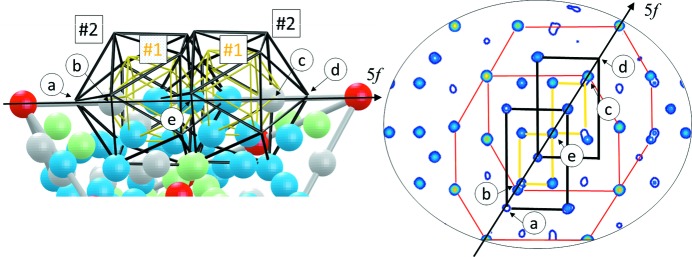
The formation of the *a* linkage in the asymmetric part of the OR. The #1 shell (yellow line) and the #2 shell (black line) of linked clusters are shown. The position ‘a’ (‘d’) is unoccupied due to the close vicinity of the atom ‘b’ (‘c’). The site ‘a’ shows some non-zero electron density (right) but in the refined model this site is unoccupied. The position ‘e’ is a potential split position created as an overlay of two neighbouring #1 shells. The RTH shell was not plotted in the atomistic model as it would make the picture indistinct.

The short linkages create split atomic positions that have to be dealt with. In our model, because of the way the atomic positions were identified by looking for the maxima in the electron density, one of two split atomic maxima can be unoccupied as the algorithm removes one maximum if two are too close to each other. Those positions are *e.g.* the atoms belonging to the icosahedron from the #2 icosahedral shell of clusters located on the edges of rhombohedra (marked ‘a’ and ‘d’ in Fig. 8 ▸). That does not worsen the model, but high phononic ADPs for such positions are expected (see Section 8.3). The site ‘a’ is unoccupied because it would be too close to the site ‘b’. The same reasoning applies to the site ‘d’. The sites ‘b’ and ‘c’ exhibit a substantial freedom of movement along a fivefold direction, that is reflected in the electron-density map. That is the phason flip site. Due to the steric condition the site ‘a’ can be unoccupied, as it is presented, or occupied depending on how far the atom ‘b’ is from the cluster centre. We can see that in the electron-density map the site ‘a’ could be partially occupied or occupied by a light element, but due to the low electron density it remained empty in the model. In contrast to the site ‘a’, the site ‘d’ shows no indication of the atom (Fig. 8 ▸, right).

There is another site that deserves some attention. The site ‘e’ is shared by the #1 shell of both interacting clusters. The question is whether this site creates a short distance that should be filled by a split atom. In the electron-density map, there is no trace of smearing along the fivefold direction. However, due to the manoeuvrability of the sites ‘c’ and ‘b’, there is a possibility that the site ‘e’ will be able to glide along the fivefold axis. Nevertheless, in the model it is occupied by one atom.

The other intriguing question is whether the cluster centre is occupied by an atom or not. In our model the occupied centre site is created by the existence of the *a* linkage. The atom occupying the node of the icosahedron from the #2 icosa­hedral shell is located right where the centre of the nearby cluster is located. That is very well represented in Fig. 8 ▸, both in a refined structure model and an electron density obtained from the *ab initio* phasing. Only the clusters located on the long body-diagonal of the AR do not exhibit the occupied centre as they are connected by the *c* linkage and do not exhibit any *a* linkage. However, they are linked by the short *b* linkage with neighbouring clusters.

**Figure 9:**
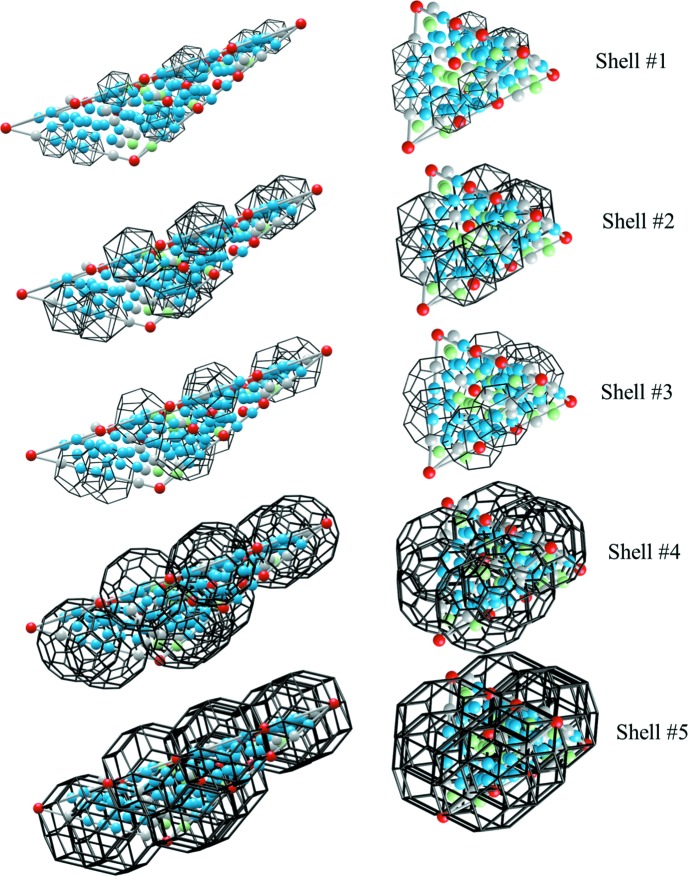
All the shells of the Bergman cluster plotted for the asymmetric part of the AR (left) and OR (right). The colour scheme for atoms is the same as that in Fig. 4 ▸. Each of the atoms belongs to one of the cluster’s shells.

Our refined model shows all the features that were also found in the *ab initio* structure solution. At this point we would like to state that at the very early stage of the iQC structure solution, there was an attempt at constructing the iQC with two types of RTH clusters only: a small one and a big one (Audier *et al.*, 1988 ▸). The model was derived for the cubic *R*-Al_5_CuLi_3_ approximant of the AlCuLi QC. The crucial difference with respect to our approach is we do not include the second, smaller RTH in-between large RTH clusters. All the shells of the RTH covering derived from our model can be seen in Fig. 9 ▸ for the asymmetric part of the rhombohedra.

Ten sites in the asymmetric part of the AR and seven sites in the OR were found to host the RTH cluster. Those sites are listed in Table 1 ▸. To create this list, we have used the known relation for τ scaling: every number can be expressed as equal to 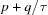, where 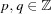. The list shows (*p*, *q*) values of the sites in the rhombohedra.

**Figure 10:**
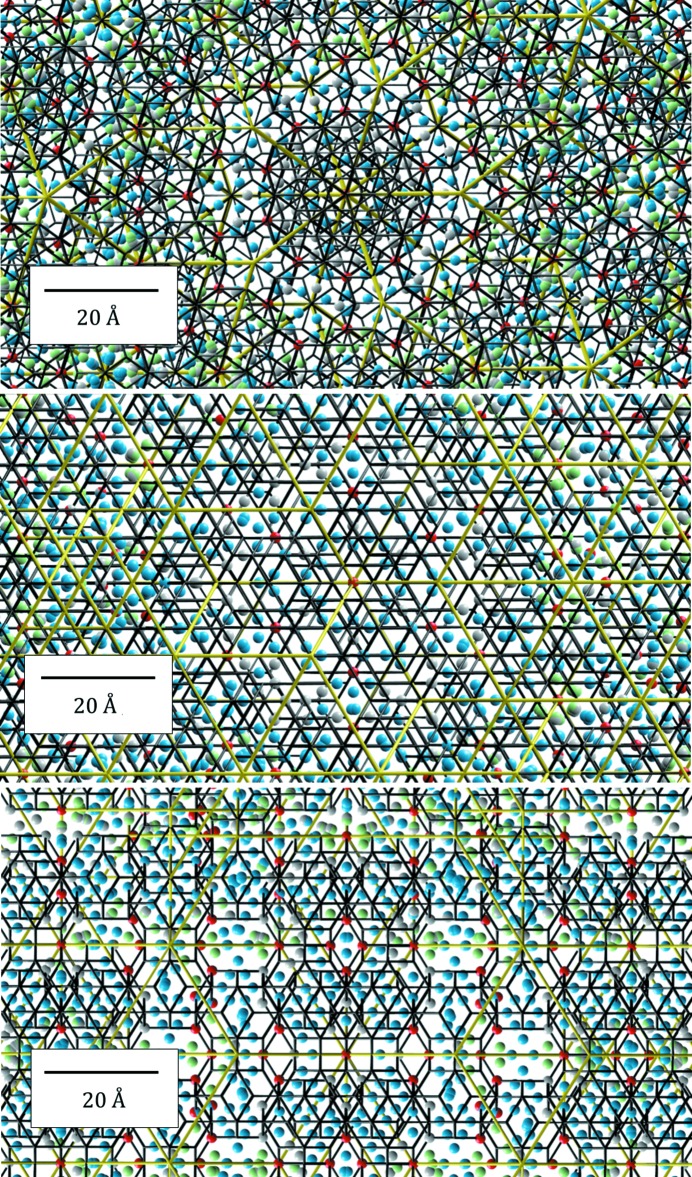
The ZnMgTm iQC structure projected along fivefold (top), threefold (middle) and twofold (bottom) directions. The thickness of the projected structure is equal to 2 Å. Black bonds define the frame of the RTH cluster, whereas the yellow bond indicates the edges of the 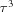-inflated rhombohedra in the AKNt.

To illustrate the refined structure, in Fig. 10 ▸ the 2D projections of the atomic arrangement along two-, three- and fivefold directions are shown. The RTH cluster covering and the AKNt are plotted over the generated atomic sites for guidance. Since the cut through the AKNt perpendicularly to a fivefold direction is known to form a Penrose tiling, it is not surprising the fivefold section exhibits a similar atomic arrangement to that of the decagonal QCs.

## 7. The higher-dimensional model   

**Figure 11:**
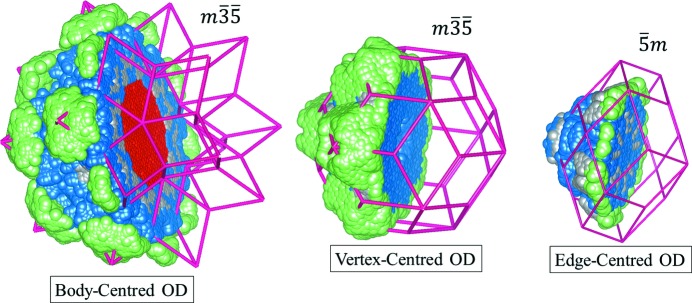
Three ODs of the ZnMgTm iQC lifted to the 6D space: the OD_B_ (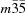), OD_V_ (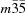) and OD_E_ (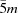). The Tm is accumulated only in the OD_B_. The empty centre of OD_V_ is reproduced very well in the present model.

The traditional approach, with 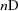, involves modelling the ODs located in the internal space, being orthogonal to the physical space. Our model, despite being based on the real space, can still be lifted to the 6D space to compare our result with previous attempts on the Bergman-type QC. In order to lift the structure to 6D a large portion (>500 000) of the atomic positions were generated. All the positions were represented as 6D vectors, where the physical space coordinates were derived from the structure itself and the internal space coordinates were assigned 0. It is due to the principle of the section method where the physical space coordinate is generated as an intersection of the physical space with the OD. That always occurs for the internal space coordinates equal to 0. After multiplying the 6D vector of each atom by the inverse projection matrix 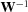, the coordinates in the 6D space were found. The coordinates were then reduced to one 6D unit cell by the modulo 1 operation. Every position of the generated structure was then assigned to the corresponding OD. The assignment is not deterministic because of the phason flip sites that allow for an atom to be ascribed to two different ODs with equal probability. The recreated ODs are plotted in Fig. 11 ▸ and compared with their equivalents coming from the simple decoration model (Elser & Henley, 1985 ▸). The inner part of the OD_B_ generates Tm atoms in the structure. Tm is only located in that OD which corresponds well with the fivefold section plotted in Fig. 2 ▸. In the OD_V_ the inner part is empty which also corresponds very well to the plot in Fig. 2 ▸ obtained from the *ab initio* phasing procedure. Those positions are related to the cluster centres in the body-diagonal of the AR. The OD_E_ is located at the low-symmetry site (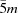) which is also recreated in our model. The recreated ODs resemble those for the simple decoration model in terms of size and shape, but the details are different. For instance, we can see unoccupied sites along the threefold direction in the OD_V_ and OD_B_. That fine structure corresponds to fine-tuning carried out by Yamamoto to solve the structure of the AlCuLi iQC (Yamamoto, 1992 ▸). He additionally modified the simple decoration model by removing the atom from the 12-fold vertices (empty centre in OD_B_), and also removing some atoms inside the rhombic dodecahedron lying on the mid-edge positions and putting atoms in off-edge-centre positions. However, the fine details of the ODs are different, *e.g.* we observe an aggregation of Mg atoms nearby fivefold vertices in the OD_V_, which does not occur in Yamamoto’s model, possibly due to its idealized decoration of off-centre mid-edge positions. In addition, our model is significantly more ordered chemically.

**Figure 12:**
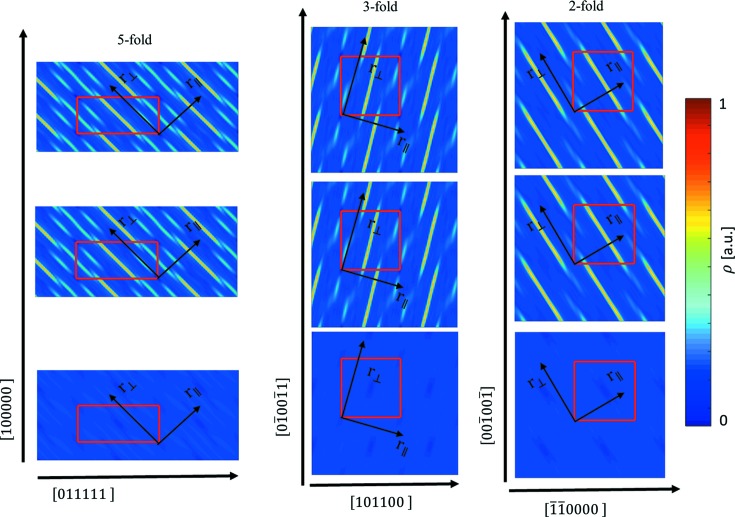
The high-symmetry 2D sections through the 6D space resulting from the Fourier transform of the experimental diffraction pattern (top), our model after the refinement (middle) and the differential electron-density map (bottom). The small residual density validates the correctness of the model. The red rectangle shows the 6D unit cell.

The last test of our model was the comparison between the calculated high-symmetry sections through the 6D space. The phases obtained from the refined structure allowed us to calculate the electron-density map using the experimental diffraction amplitudes and ones recovered from the model (Fig. 12 ▸). By calculating the residual electron density 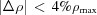 we can conclude that our model does not miss any significant amount of atoms in the structure. The comparison with other Bergman QCs is impossible since no other contemporary model of this QC exists.

## 8. Discussion   

### 8.1. Tiling approach versus cluster model   

The model of the ZnMgTm Bergamn iQC was constructed in the tiling-and-decoration scheme, where two golden rhombohedra of the AKNt were chosen as building blocks of the model. This alternative to the cluster approach definition of the model was previously proposed based on the structure refinement of the 2/1 cubic approximant of the AlZnMg QC (Lin & Corbett, 2006 ▸). Unfortunately, the model was never finished, possibly due to the problem of finding a unique atomic decoration of AR and OR. We dealt with this problem by proposing 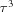-inflated rhombohedra as fundamental building blocks of the structure. The question that could be asked is whether such an approach is justified. In fact, that question is much deeper. So far, all the structure solutions of QCs make a presumption that a long-range order is well approximated by the tiling. Sometimes, the assumption is explicit, when *e.g.* the structure of the decagonal QC is built upon the Penrose tiling. In other situations, like in the case of iQCs, this assumption is hidden when only the subset of the 12-fold vertices of the AKNt is used to define the locations of cluster centres. Either way, the property of tiling is exploited, even though it is still unknown whether a particular tiling well characterizes the structure of the QC. There has been an attempt to generalize the tilings during the structure refinement of the decagonal QCs (Chodyn *et al.*, 2015 ▸), but such a work does not exist for the AKNt.

It is difficult to say whether the cluster approach contradicts or is equivalent to the tiling approach in the context of the iQC. The solved structure exhibits the *a* linkage and the short *b* linkage which are absent in the cluster model. That would mean those two approaches cannot be equivalent. But what if the interstitial structure in the cluster model could be interpreted in terms of the clusters but with different atomic decoration? It is already known that atoms in the Tsai-type QC model form a Bergman cluster in the interstitial part (Takakura *et al.*, 2007 ▸). If RTH clusters were assigned to cover the interstitial part, the additional linkages other than the known *b* and *c* ones, that were observed in our study, could naturally arise. We believe that a new model of the Tsai-type QC, based on the AKNt decoration, would be beneficial for a better understanding of the structure of iQCs.

### 8.2. RE distribution   

The location of Tm atoms in the present model is particularly important. The main reason is that the magnetic properties of the Zn–Mg–RE QC originate from the 4*f* electrons of the RE ions, interacting via the indirect Ruderman–Kittel–Kasuya–Yoshida exchange (Ruderman & Kittel, 1954 ▸; Kasuya, 1956 ▸; Yosida, 1957 ▸). So far, all the research conducted on the Zn–Mg–RE QCs shows the spin-glass-like behaviour at low temperature and no clear indication of the long-range order has been reported. The nature of the frozen state is still under discussion (Goldman, 2014 ▸).

One of the attempts at theoretical investigation of the magnetic ordering in the FCI QCs was made by Sato (2005 ▸), who proposed a model where the RE atoms form the network of edge-sharing dodecahedra with an edge length of 5.5 Å. However, the dodecahedral spin object does not explain why the spin correlation is terminated at the finite length. From the geometric point of view, there is no reason why two adjacent dodecahedra should behave as isolated objects which was indicated by the author. Even though the question remains open, the dodecahedron as a spin object at least explains the magnetic diffuse scattering in the neutron diffraction pattern.

Recently, a FCI Bergman QC was investigated in ZnMgHf in terms of the local atomic arrangement, especially dedicated to the positions of the Hf elements (Buganski *et al.*, 2019 ▸). Similar to RE elements, Hf seems to occupy the #5 RTH shell of the Bergman cluster. In that study, the Hf elements are not only located in the threefold vertices of the RTH cluster, forming the dodecahedron, but also in the fivefold vertices of the second cluster, forming the icosahedron. In principle, the existence of two types of clusters, differing by the preferential occupation of the high-symmetry sites by Hf, is the reason the face-centred ordering is realized. We believe that this new result could benefit the modelling of the magnetic properties in Bergman QCs; however, the complete structure solution of the FCI QC is still missing.

**Figure 13:**
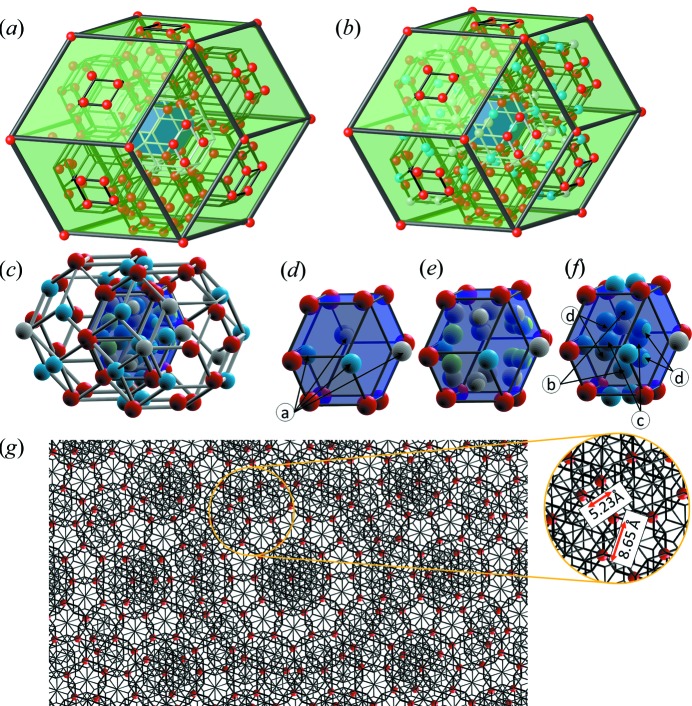
The atomic decoration of the dodecahedral motif in the refined structure of the ZnMgTm Bergman iQC. The RE atoms in (*a*) and all the atoms in (*b*) occupying the RTH’s #5 shell are shown. The inner blue dodecahedron is 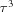 times smaller with respect to the green one showing the scaling property; (*c*) the intersection of the RTH clusters inside the dodecahedron, forming a short *b* linkage. The atoms occupying the vertices of the triacontahedron and all the atoms in the small dodecahedron are shown. In (*d*), (*e*), (*f*) the atomic decoration of the small dodecahedra, created by the intersection of two RTH clusters along a short *b* linkage, is shown. (*d*) shows the outer atoms, (*e*) shows Mg and mixed occupation atoms and (*f*) shows Zn atoms inside the small dodecahedron; (*g*) the piece of the structure projected along a fivefold axis presenting the distribution of Tm atoms. The 1 Å depth is shown. The black bonds determine the frame of the RTH cluster.

The result of the refinement of the PI ZnMgTm QC is in some way even more complex in terms of the Tm element distribution than what is already known for the FCI phase. In Fig. 13 ▸ the distribution of atoms in the rhombic dodecahedron is shown, focusing on the Tm atoms. All the Tm atoms are on the vertices of the RTH [Fig. 13 ▸(*a*)]; however, not all the vertices are occupied by Tm atoms. It is difficult to formulate a rule stating which site of the cluster is occupied. Both threefold and fivefold vertices in the same cluster can be occupied at the same time, and therefore the exclusion rule valid for the FCI does not apply. The #5 shell of the RTH cluster, besides Tm atoms, is occupied by Zn and Zn/Tm atoms. Some vertices are occupied by the Zn atoms solely. Such a non-unique decoration of cluster sites originates from clusters overlapping along more than *b* and *c* linkages. The number of local environments of clusters grows when the *a* and the short *b* linkages are additionally allowed. Therefore, the cluster does not represent well the local chemical order.

The rhombic dodecahedron can be decomposed into two ARs and two ORs. Inside the shown dodecahedron the short *b* linkage [white clusters in Figs. 13 ▸(*a*), 13 ▸(*b*) and 13 ▸(*c*)] originates from two adjacent ORs, contributing to formation of the small rhombic dodecahedron. In Figs. 13 ▸(*d*)–13 ▸(*f*) the decoration of the small dodecahedron, resulting from the intersection of two RTH clusters, is shown. Tm atoms are gathered on the ring occupying the vertices of the dodeca­hedron but the sites that lie on the plane perpendicularly to the linkage direction are occupied by Zn and mixed Zn/Tm atoms [sites ‘a’ in Fig. 13 ▸(*d*)]. These sites are generated by the overlay of the RTH and inner icosahedron resulting in a split atomic position, as was mentioned during the discussion of Fig. 3 ▸. The Zn/Mg and Mg atoms are located on the surface of the rhombic face, creating a split position along the twofold direction Those sites are generated by the #4 soccer-ball polyhedral shell. Zn atoms form a hexagonal ring inside the rhombic dodecahedron perpendicular to a threefold direction [Fig. 13 ▸(*f*)]. In this ring sites ‘b’ come from the nodes of the #2 icosahedral shell, the sites ‘c’ are generated by the #3 dodeca­hedral shell and the sites ‘d’ are once again generated by the #4 soccer-ball polyhedral shell.

In Figs. 13 ▸(*a*) and 13 ▸(*b*) not all the RTH clusters are plotted. For the sake of simplicity, only the clusters for which one of the faces lies on the face of the dodecahedron, forming a rhombus of Tm atoms, are plotted. In Fig. 13 ▸(*g*) the 2D projection, along a fivefold axis, of the Tm atoms in the structure is shown. The frame of the RTH clusters is plotted for guidance, to indicate each of the atoms belong to the vertex of the #5 shell. The Tm–Tm distance is equal to the edge length of the RTH. Even though it was estimated to be 5.13 Å, the Tm–Tm distance in the presented cut through the structure is equal to 5.23 Å between the nearest-neighbour and 8.65 Å between the second-nearest-neighbour atoms.

### 8.3. Atomic ADPs and disorder   

**Figure 14:**
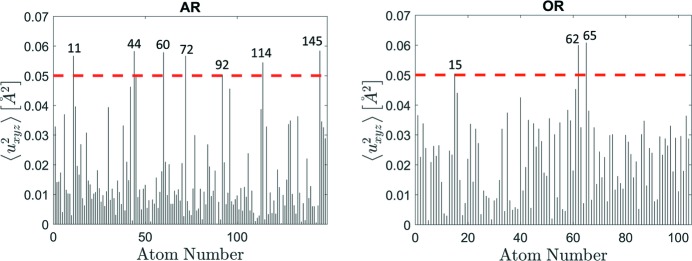
The mean-square displacement parameters (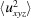) plotted for atoms in the asymmetric part of the AR (left) and OR (right). The cut-off value of 0.05 Å^2^ is shown (red dotted line). The labels of atoms that exceed the threshold are given.

The refined structure model of the ZnMgTm iQC shows rather small chemical and positional disorders, compared with known Bergman QC approximants (Gómez *et al.*, 2008 ▸; Lin & Corbett, 2006 ▸). The maximal atomic mean-square displacement parameter 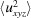, related to *B* factors according to the formula 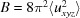, found in the refined structure is equal to 0.061 Å^2^. It is estimated for one of the Tm atoms in the OR. Such a value is still almost three times lower than for the Zn18 position in the ZnMgZr 1/1 cubic approximant (Gómez *et al.*, 2008 ▸). In Fig. 14 ▸ the atomic mean-square displacement was plotted for each atom from the asymmetric part of the AR and OR. We decided to thoroughly analyse the origin of the large displacement parameter for atoms that exhibit 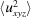 > 0.05 Å^2^. The threshold was chosen arbitrarily, for practical reasons, just to present the idea of why the large displacement occurs.

Seven atoms from the AR satisfy this condition: 11, 44, 60, 72, 92, 114 and 145. Labels correspond to numbers given in Table S1 in the supporting information. All the above-listed atoms experience rather significant displacement in the course of the structure refinement. The shift is about 1 Å from the position in the initial model. In all cases, such a substantial rearrangement is induced by the existence of the split atomic site which was not an assumption of our model and instead only one fully occupied atom was considered. Atom 11 is Zn located at the face of the AR. It is generated by the #4 soccer-ball polyhedral shell, that was indicated in Fig. 4 ▸ to exhibit a considerable disorder. Atoms 60 and 72 are Zn atoms generated by the #3 dodecahedral shell. The nodes of the dodecahedron become disordered due to the formation of the *a* linkage. Atoms 92 and 114 are Zn generated by the #1 icosahedral shell. The strong displacement of the positions is justified by the *a* linkage formed by RTH clusters. The behaviour is similar to that which was presented for the sites ‘a’ and ‘b’ in Fig. 8 ▸. Due to the absence of the atom in the site ‘a’, the atom ‘b’ has space for mobility along a fivefold direction. In the case of atom 92 such mobility occurs along the long body-diagonal of the AR. Atoms 44 and 145 are Tm atoms located in the vertices of the AR. Their potential manoeuvrability is caused by the absent atom in sites ‘a’ and ‘d’ in Fig. 8 ▸. In addition, these atomic positions were fixed during the structure refinement in the vertices of the AR. Therefore, they might not be at their optimal position.

The OR has fewer atoms that are significantly disordered in terms of ADPs. Only three atoms satisfy the above-mentioned condition: 15, 62 and 65. Atom 15 is Zn, generated by the #2 icosahedral shell, forming the *a* linkage. Atoms 62 and 65 are Tm atoms lying on the face of the OR, sharing the same face as the #5 shell. The positions of Tm atoms can be affected by rather strongly disordered Mg atoms, generated by the #4 soccer-ball polyhedral shell, which surround atom 62. Any disturbance of the #4 shell will affect the #5 shell. Those two Tm atoms show the two largest mean displacement parameters: 0.060 Å^2^ for atom 62 and 0.061 Å^2^ for atom 65. Also, three extreme Tm—Tm bond lengths occur in this patch: Tm_62_—Tm_38_ = 4.3 Å, Tm_62_—Tm_65_ = 5 Å and Tm_38_—Tm_65_ = 5.8 Å.

It can be speculated as to what is better for the structure refinement – the explicit split atomic position or a single atom with a large displacement parameter. It is a difficult question especially in the context of QCs. Since the number of local environments in QCs is significantly larger than those in approximant crystals, principally in the Bergman QC where additional linkages are identified, it could be extremely tedious to assign all the split atomic positions correctly. Therefore, we decided not to introduce any more split positions and accept slightly larger phononic ADPs.

Besides positional disorder, the structure model displays some chemical disorder. The mixed atoms predominantly occupy the positions on the edges of rhombohedra, generated by the #1 icosahedral shell, where the *a* linkage is formed. In this case the Zn/Mg mixed site is generated. Additional mixing occurs for atoms on the dodecahedral shell, *e.g.* atoms 131 and 121 of the AR, forming the *c* linkage. The site is shared with the vertex position of the triacontahedral shell. Those positions are mixed Zn/Mg with trace amounts of Tm atoms. It could however be an artefact of the refinement since the number of peaks to parameters ratio is low. A number of mixed atoms also occur for the sites shared by the soccer-ball polyhedra and the innermost icosahedron in the short *b* linkage. Such a site is *e.g.* position 49 of the AR being occupied by mixed Zn/Tm in a ratio 0.63/0.37. At this point we cannot determine whether or not there is a clear selectivity rule for chemical species to occupy a specific position in the shells of clusters. However it is evident that mixing of the chemical species occurs as a consequence of the overlay of the atomic shells of neighbouring clusters.

The partially occupied sites are atoms 104 and 101 from the OR, that are generated by the #4 soccer-ball polyhedral shell, and 146, 147 and finally 148 from the AR. Atom 146 forms a phason flip site with atom 55. Atom 147 generates a close distance with atom 145. It is therefore not surprising that low occupancy of site 147 with probability 0.15 is obtained.

Finally, we would like to comment on the magnitude of the phason disorder. The phason correction was made by the general Debye–Waller formula with the phasonic ADP equal to 1.44 Å^2^. That value is rather low compared with that for other known iQCs (Yamada *et al.*, 2016 ▸, 2017 ▸; Takakura *et al.*, 2007 ▸), which is around 3.5 Å^2^. Out of curiosity, the phasonic ADP was forcefully set to 0 Å^2^, to check how much the value of the *R* factor would change. To our surprise, the *R* factor was almost unchanged and was equal to 9.9%. That is a very important result. The phasonic disorder is known to be correlated to single atomic jumps or the frozen lattice disorder associated with the tiling. Single atomic jumps to some extent can be modelled by introducing partial occupancies. However, nothing can be done about the lattice part. The lattice randomization by phasons can only be modelled by the phasonic Debye–Waller factor (Buganski *et al.*, 2017 ▸, 2019 ▸). Since our result stays the same when the phasonic ADP is neglected, that means the tiling is correctly chosen. That could mean the AKNt describes very well the structure of the Bergman QC.

## 9. Conclusions   

The structure model for the PI ZnMgTm QC is proposed based on the atomic decoration of the two rhombohedra in the Ammann–Kramer–Neri tiling. The rhombohedra used in the model are 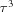 inflated in comparison with standard lattice size. The inflation allows us to avoid the problem of an ambiguous decoration of rhombohedra in the interstitial part of the structure which exists in a cluster-based model. The ambiguity was the main obstacle in the construction of the occupation domains for the structure modelling in a 6D embedding. For the structure refinement, we rejected the higher-dimensional approach in favour of the real-space approach. Since the structure modelling was based on the real-space atomic decoration of rhombohedra, it is redundant to lift the structure to 6D just for the refinement.

The iQC was classified as the Bergman QC based on the inner structure of the rhombic triacontahedral cluster. The 3D electron-density map, reconstructed after phasing with *Superflip*, shows four possible linkages between rhombic triacontahedral clusters. Apart from known *b* and *c* linkages, there are short *b* and *a* linkages. The interaction of atoms due to the existence of additional linkages creates split atomic positions in the high-symmetry sites of the structure and a few instances of large ADPs. All the characteristic features observed in the *ab initio* structure solution are well represented in the refined structure.

The refinement resulted in a very good agreement of the calculated structure factor with the experimental diffraction amplitudes, measured by the crystallographic *R* factor equal to 9.8% with the reflections-to-parameters ratio of about 3.9. The model shows an inherent chemical mixing of atoms which is commonly observed for ZnMg-RE QCs and approximants.

The analysis of the refined structure in terms of the Bergman cluster distribution shows two major novelties:

(i) A whole structure can be represented as a covering by rhombic triacontahedral clusters. All the atoms are generated by the vertices of five shells of the Bergman cluster expanded with the outermost shell of the rhombic triacontahedron. No interstitial part of the structure must be distinguished, in contrast to what was proposed for binary CdYb QCs.

(ii) Apart from the *b* and *c* linkages given by the 12-fold sphere packing geometry, the short *b* and *a* linkages are present in the model. The linkage along a fivefold direction is primarily responsible for the occurrence of the occupied cluster centres observed in the electron-density map. The node of the #2 icosahedral shell interpenetrates the central position of the neighbouring cluster and is perceived as a lonely atom inside the cluster. The *a* linkage does not introduce short interatomic distances as the sites that are in close vicinity to each other become split atomic sites. In reality, those sites are creating space for the occurrence of the phason flips.

The main question is whether the proposed model, with the cluster covering, could be used for two other families of iQCs: Tsai and Mackay. Before the attempt at structure modelling is made we cannot answer this question. To the best of our knowledge, only the Bergman QC demonstrates such a peculiar atomic structure.

When it comes to the RE atom distribution, it is difficult to tell unambiguously which cluster sites are occupied by this element. In principle, the Tm element is located at the vertices of the triacontahedron but due to the short *a* linkage and the *c* linkage the inner dodecahedral shell can also be occupied by Tm atoms. To our knowledge, the occupation of the triacontahedron by Tm depends on the local arrangement of the RTH clusters but the number of such local environments is much greater than those of the standard cluster-based model with *b* and *c* linkages only. It is therefore much more convenient to use the golden rhombohedra than cluster to determine where the atoms in the structure really are.

## Supplementary Material

Crystal structure: contains datablock(s) ZnMgTm_Acute_asymmetric, ZnMgTm_Obtuse_asymmetric. DOI: 10.1107/S2053273319017339/ae5079sup1.cif

Diffraction data used for structure solution and refinement. DOI: 10.1107/S2053273319017339/ae5079sup2.txt

List of parameters describing the structure model of ZnMgTm iQC. DOI: 10.1107/S2053273319017339/ae5079sup3.pdf

CCDC references: 1974795, 1974796

## Figures and Tables

**Table 1:** RTH cluster positions in two rhombohedra: AR and OR The notation gives (*p*, *q*) values of the reduced coordinates of cluster centres. For example, the position *r* of the cluster centre is equal to 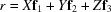, where 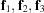 are vectors spanning edges of the rhombohedra. For the AR those vectors are 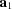, 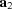, 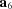, respectively, and for the OR 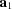, 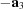, 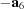.

Rhombohedron type	*X*	*Y*	*Z*
AR	(0, 1)	(0, 1)	(0, 1)
	(1, −1)	(1, −1)	(1, −1)
	(0, 1)	(0, 0)	(0, 0)
	(1, −1)	(0, 0)	(0, 0)
	(2, −2)	(2, −3)	(2, −2)
	(−1, 3)	(−1, 2)	(−1, 2)
	(1, 0)	(0, 0)	(0, 1)
	(1, 0)	(0, 0)	(1, −1)
	(1, 0)	(1, −1)	(1, 0)
	(1, 0)	(0, 1)	(1, 0)
OR	(0, 1)	(0, 0)	(0, 0)
	(1, −1)	(0, 0)	(0, 0)
	(1, 0)	(0, 0)	(0, 1)
	(1, 0)	(0, 0)	(1, −1)
	(1, 0)	(1, −1)	(1, 0)
	(1, 0)	(0, 1)	(1, 0)
	(0, 1)	(1, −1)	(0, 1)
