# Supplementary material for: The atomic structure of the Bergman-type icosahedral quasicrystal based on the Ammann–Kramer–Neri tiling
Source: Acta Crystallogr A Found Adv. 2020 Feb 11;76(Pt 2):180–96. doi: 10.1107/S2053273319017339 (PMC7053224; doi:10.1107/S2053273319017339)
Supplement: Supplementary file 3 [file a-76-00180-sup3.pdf]

FOUNDATIONS
ADVANCES

Volume 76 (2020)

Supporting information for article:

The atomic structure of the Bergman-type icosahedral quasicrystal based on the Ammann-Kramer-Neri tiling

Ireneusz Buganski, Janusz Wolny and Hiroyuki Takakura

In Table S1 we submit the list of the refined parameters. Coordinates x, y and z are given in a rhombohedron-spanning base, what means real-structure **r** coordinate vector is obtained as follows: $r = X\mathbf{f}_1 + Y\mathbf{f}_2 + Z\mathbf{f}_3$, where $\mathbf{f}_1, \mathbf{f}_2, \mathbf{f}_3$ are vectors spanning edges of the rhombohedra. For the AR those vectors are $\mathbf{a}_1$, $\mathbf{a}_2$, $\mathbf{a}_6$ respectively and for the OR $\mathbf{a}_1$, $-\mathbf{a}_3$, $-\mathbf{a}_6$. The letter 'L' stands for an acute rhomb and the letter 'S' stands for a obtuse rhombus. SOF defines site of fraction: 1 means the position is fully occupied, 0 means there is no atom there. The column labeled „Fraction" gives the ratio of atom inside a rhombus depending on it position in a rhombus f. i. atom on the edge is only in half inside a rhombus. Variable $\langle u^2{}_{xyz} \rangle$ defines mean atomic displacement.

Table S1 The list of parameters describing the structure model of ZnMgTm iQC.

Unit Type	Nr	x	y	z	p(Zn)	p(Mg)	p(Tm)	SOF	$\langle u^2{}_{xyz} \rangle$ [Å^2]	Fraction
L	1	1.0000	1.0000	1.0000	0.0000	0.0000	1.0000	1.0000	0.032873	0.0083
L	2	0.8511	0.8511	0.8511	1.0000	0.0000	0.0000	1.0000	0.014136	0.166667
L	3	1.0000	0.8490	0.8489	1.0000	0.0000	0.0000	1.0000	0.014304	0.5
L	4	0.7654	0.7655	0.7654	0.0000	0.0000	1.0000	1.0000	0.017362	0.166667
L	5	1.0000	0.7656	0.7656	0.6017	0.0000	0.3983	1.0000	0.004038	0.25
L	6	0.7065	0.7087	0.7066	1.0000	0.0000	0.0000	1.0000	0.03697	0.166667
L	7	0.7361	0.6186	0.6186	1.0000	0.0000	0.0000	1.0000	0.011544	0.5
L	8	0.8503	0.6170	0.6170	1.0000	0.0000	0.0000	1.0000	0.010316	0.5
L	9	1.0000	0.6357	0.6096	0.0000	0.0000	1.0000	1.0000	0.010224	0.25
L	10	0.7298	0.5490	0.5490	1.0000	0.0000	0.0000	1.0000	0.002974	0.5
L	11	1.0000	0.5634	0.5428	1.0000	0.0000	0.0000	1.0000	0.05667	0.5
L	12	0.5258	0.5258	0.5250	1.0000	0.0000	0.0000	1.0000	0.039609	0.166667
L	13	0.6961	0.4836	0.4836	1.0000	0.0000	0.0000	1.0000	0.019586	0.5
L	14	0.8493	0.4787	0.4787	1.0000	0.0000	0.0000	1.0000	0.016645	0.5
L	15	1.0000	0.4931	0.4333	1.0000	0.0000	0.0000	1.0000	0.026908	0.5
L	16	0.4688	0.4688	0.4688	0.0000	0.0000	1.0000	1.0000	0.0087	0.166667
L	17	1.0000	0.3843	0.3843	0.0000	0.0000	1.0000	1.0000	0.006281	0.25
L	18	0.3768	0.3635	0.3462	1.0000	0.0000	0.0000	1.0000	0.030785	0.166667

L	19	0.4971	0.3820	0.3819	1.0000	0.0000	0.0000	1.0000	0.014663	0.5
L	20	0.6149	0.3837	0.3797	1.0000	0.0000	0.0000	1.0000	0.013353	0.5
L	21	0.7679	0.3818	0.3740	0.0000	0.0000	1.0000	1.0000	0.008512	0.5
L	22	0.4948	0.3128	0.3128	1.0000	0.0000	0.0000	1.0000	0.010262	0.5
L	23	0.7308	0.3091	0.3088	1.0000	0.0000	0.0000	1.0000	0.010794	0.5
L	24	0.2346	0.2355	0.2350	0.0000	0.0000	1.0000	1.0000	0.018126	0.166667
L	25	0.4707	0.2407	0.2407	0.6863	0.0000	0.3137	1.0000	0.012074	0.5
L	26	0.6102	0.2398	0.2398	1.0000	0.0000	0.0000	1.0000	0.007521	0.5
L	27	0.7327	0.2384	0.2384	1.0000	0.0000	0.0000	1.0000	0.009739	0.5
L	28	0.8576	0.2329	0.2329	1.0000	0.0000	0.0000	1.0000	0.006094	0.5
L	29	1.0000	0.2405	0.2405	1.0000	0.0000	0.0000	1.0000	0.011048	0.25
L	30	0.1530	0.1533	0.1529	1.0000	0.0000	0.0000	1.0000	0.039365	0.166667
L	31	0.7597	0.1483	0.1483	0.3929	0.0000	0.6071	1.0000	0.008154	0.5
L	32	0.2680	0.1461	0.1402	1.0000	0.0000	0.0000	1.0000	0.011936	0.5
L	33	0.3845	0.1610	0.1295	1.0000	0.0000	0.0000	1.0000	0.00872	0.5
L	34	0.5213	0.1452	0.1452	1.0000	0.0000	0.0000	1.0000	0.003104	0.5
L	35	0.7120	0.0863	0.0863	1.0000	0.0000	0.0000	1.0000	0.014675	0.5
L	36	0.2637	0.0727	0.0727	1.0000	0.0000	0.0000	1.0000	0.010787	0.5
L	37	0.4981	0.0730	0.0730	1.0000	0.0000	0.0000	1.0000	0.006758	0.5
L	38	0.0000	0.0000	0.0000	0.0000	0.0000	1.0000	1.0000	0.0332	0.0083
L	39	0.2340	0.0000	0.0000	0.5331	0.0000	0.4669	1.0000	0.004593	0.1
L	40	0.3869	0.0000	0.0000	1.0000	0.0000	0.0000	1.0000	0.021	0.1
L	41	0.5042	0.0000	0.0000	1.0000	0.0000	0.0000	1.0000	0.014783	0.1
L	42	0.6021	0.0000	0.0000	1.0000	0.0000	0.0000	1.0000	0.046273	0.1
L	43	0.8357	0.0000	0.0000	0.4471	0.4293	0.1236	1.0000	0.00122	0.1
L	44	1.0000	0.0000	0.0000	0.0000	0.0000	1.0000	1.0000	0.05824	0.075
L	45	1.0000	0.1500	0.1969	1.0000	0.0000	0.0000	1.0000	0.049964	0.5
L	46	0.9278	0.7039	0.7271	0.5000	0.5000	0.0000	1.0000	0.009076	1
L	47	0.9175	0.3365	0.3368	1.0000	0.0000	0.0000	1.0000	0.011649	0.5
L	48	0.8529	0.6191	0.7349	1.0000	0.0000	0.0000	1.0000	0.004927	1

L	49	0.6176	0.5061	0.6176	0.6313	0.0000	0.3687	1.0000	0.011912	0.5
L	50	0.6213	0.3836	0.4987	1.0000	0.0000	0.0000	1.0000	0.013214	1
L	51	0.8419	0.3622	0.4812	1.0000	0.0000	0.0000	1.0000	0.005434	1
L	52	0.3827	0.2660	0.3827	1.0000	0.0000	0.0000	1.0000	0.008038	0.5
L	53	0.3835	0.1527	0.2648	1.0000	0.0000	0.0000	1.0000	0.000855	1
L	54	0.6180	0.1175	0.2307	0.8343	0.0000	0.1657	1.0000	0.008275	1
L	55	0.8091	0.0000	0.1207	0.3806	0.3419	0.2775	1.0000	0.006894	0.5
L	56	0.6190	0.0000	0.1159	1.0000	0.0000	0.0000	1.0000	0.015657	0.5
L	57	0.8489	0.7359	0.8608	1.0000	0.0000	0.0000	1.0000	0.005441	0.5
L	58	0.8595	0.2310	0.3554	1.0000	0.0000	0.0000	1.0000	0.010848	1
L	59	0.8506	0.1067	0.2459	1.0000	0.0000	0.0000	1.0000	0.017778	1
L	60	0.8065	0.4662	0.6012	1.0000	0.0000	0.0000	1.0000	0.05784	1
L	61	0.6102	0.2402	0.3825	1.0000	0.0000	0.0000	1.0000	0.020974	1
L	62	0.3871	0.0000	0.1519	1.0000	0.0000	0.0000	1.0000	0.009769	0.5
L	63	0.6877	0.5045	0.6884	1.0000	0.0000	0.0000	1.0000	0.020141	0.5
L	64	0.6898	0.1166	0.3094	1.0000	0.0000	0.0000	1.0000	0.006825	1
L	65	0.9278	0.1162	0.3103	1.0000	0.0000	0.0000	1.0000	0.006839	1
L	66	0.1566	0.0000	0.1566	1.0000	0.0000	0.0000	1.0000	0.011243	0.5
L	67	0.9267	0.7346	0.9267	1.0000	0.0000	0.0000	1.0000	0.009881	0.5
L	68	0.9263	0.4992	0.6917	1.0000	0.0000	0.0000	1.0000	0.011715	1
L	69	0.9263	0.3549	0.5472	1.0000	0.0000	0.0000	1.0000	0.007913	1
L	70	0.5194	0.3238	0.5173	0.0000	1.0000	0.0000	1.0000	0.020522	1
L	71	0.6936	0.2659	0.4553	0.5000	0.5000	0.0000	1.0000	0.002657	1
L	72	0.5486	0.1395	0.3272	1.0000	0.0000	0.0000	1.0000	0.056672	1
L	73	1.0000	0.7663	1.0000	0.5303	0.0000	0.4697	1.0000	0.007744	0.1
L	74	1.0000	0.6118	0.8693	1.0000	0.0000	0.0000	1.0000	0.004573	0.5
L	75	0.7614	0.5257	0.7614	1.0000	0.0000	0.0000	1.0000	0.003028	0.5
L	76	0.6188	0.3861	0.6190	1.0000	0.0000	0.0000	1.0000	0.011987	0.5
L	77	0.7580	0.3842	0.6365	1.0000	0.0000	0.0000	1.0000	0.029957	1
L	78	1.0000	0.3720	0.6280	0.0000	0.0000	1.0000	1.0000	0.004578	0.5

L	79	0.7332	0.3174	0.5452	1.0000	0.0000	0.0000	1.0000	0.005226	1
L	80	0.3823	0.1548	0.3823	1.0000	0.0000	0.0000	1.0000	0.011897	0.5
L	81	0.7664	0.1424	0.3857	1.0000	0.0000	0.0000	1.0000	0.00159	1
L	82	1.0000	0.1501	0.3850	1.0000	0.0000	0.0000	1.0000	0.010919	0.5
L	83	0.4979	0.0801	0.3057	1.0000	0.0000	0.0000	1.0000	0.00665	1
L	84	0.2379	0.0000	0.2379	0.5935	0.0000	0.4065	1.0000	0.026855	0.25
L	85	0.3852	0.0000	0.2343	1.0000	0.0000	0.0000	1.0000	0.019391	0.5
L	86	0.4799	0.0000	0.2272	1.0000	0.0000	0.0000	1.0000	0.009426	0.5
L	87	0.6130	0.0000	0.2319	1.0000	0.0000	0.0000	1.0000	0.011236	0.5
L	88	0.7333	0.0000	0.2292	1.0000	0.0000	0.0000	1.0000	0.01801	0.5
L	89	0.8681	0.0000	0.2130	0.4932	0.4754	0.0313	1.0000	0.000147	0.5
L	90	1.0000	0.0000	0.2331	0.6111	0.0000	0.3889	1.0000	0.007479	0.3
L	91	0.8504	0.6168	0.8613	1.0000	0.0000	0.0000	1.0000	0.001548	0.5
L	92	0.4378	0.2199	0.4760	1.0000	0.0000	0.0000	1.0000	0.050347	0.5
L	93	0.8829	0.2637	0.4499	1.0000	0.0000	0.0000	1.0000	0.0208	1
L	94	1.0000	0.2379	0.4755	0.0000	1.0000	0.0000	1.0000	0.006703	0.5
L	95	0.2402	0.0635	0.3394	0.5000	0.5000	0.0000	1.0000	0.01057	1
L	96	1.0000	0.4989	0.7637	0.5000	0.5000	0.0000	1.0000	0.045612	0.5
L	97	0.7582	0.3917	0.7582	1.0000	0.0000	0.0000	1.0000	0.007662	0.5
L	98	0.8837	0.3770	0.7713	1.0000	0.0000	0.0000	1.0000	0.006571	1
L	99	0.8798	0.3118	0.6887	1.0000	0.0000	0.0000	1.0000	0.008387	1
L	100	0.6201	0.2343	0.6200	0.0000	0.0000	1.0000	1.0000	0.009647	0.5
L	101	0.5213	0.1410	0.5248	0.0000	1.0000	0.0000	1.0000	0.004961	0.5
L	102	0.6415	0.0773	0.4511	0.4323	0.3932	0.1744	1.0000	0.012161	1
L	103	0.6180	0.0000	0.3783	0.0000	0.0000	1.0000	1.0000	0.004508	0.5
L	104	0.7760	0.0000	0.3624	1.0000	0.0000	0.0000	1.0000	0.01251	0.5
L	105	0.8824	0.0000	0.3849	1.0000	0.0000	0.0000	1.0000	0.009183	0.5
L	106	1.0000	0.6137	1.0000	1.0000	0.0000	0.0000	1.0000	0.023861	0.1
L	107	0.8552	0.4776	0.8552	0.0000	0.0000	1.0000	1.0000	0.004587	0.5
L	108	1.0000	0.3937	0.7638	1.0000	0.0000	0.0000	1.0000	0.011252	0.5

L	109	0.8548	0.2335	0.6148	0.0000	0.0000	1.0000	1.0000	0.003821	1
L	110	1.0000	0.2356	0.6195	1.0000	0.0000	0.0000	1.0000	0.001	0.5
L	111	0.6440	0.1494	0.5266	0.0000	1.0000	0.0000	1.0000	0.002106	1
L	112	0.7626	0.1419	0.5290	0.0000	1.0000	0.0000	1.0000	0.00288	1
L	113	0.4870	0.0826	0.4865	0.5000	0.5000	0.0000	1.0000	0.03872	0.5
L	114	0.8291	0.0662	0.4610	1.0000	0.0000	0.0000	1.0000	0.054452	1
L	115	1.0000	0.0000	0.3843	1.0000	0.0000	0.0000	1.0000	0.00157	0.3
L	116	0.3814	0.0000	0.3816	0.0000	0.0000	1.0000	1.0000	0.03286	0.25
L	117	0.9273	0.5012	0.9273	1.0000	0.0000	0.0000	1.0000	0.003327	0.5
L	118	0.6914	0.2703	0.6914	1.0000	0.0000	0.0000	1.0000	0.010665	0.5
L	119	0.9251	0.1179	0.5449	1.0000	0.0000	0.0000	1.0000	0.003723	1
L	120	0.7611	0.2698	0.7611	1.0000	0.0000	0.0000	1.0000	0.007963	0.5
L	121	0.7694	0.1534	0.6376	0.4633	0.4592	0.0775	1.0000	0.003509	1
L	122	1.0000	0.4964	1.0000	1.0000	0.0000	0.0000	1.0000	0.018359	0.1
L	123	1.0000	0.3851	0.8850	1.0000	0.0000	0.0000	1.0000	0.012448	0.5
L	124	1.0000	0.1173	0.6082	1.0000	0.0000	0.0000	1.0000	0.00027	0.5
L	125	0.7631	0.0000	0.5239	0.0000	1.0000	0.0000	1.0000	0.00982	0.5
L	126	1.0000	0.0000	0.5013	1.0000	0.0000	0.0000	1.0000	0.006276	0.3
L	127	0.4638	0.0000	0.4638	1.0000	0.0000	0.0000	1.0000	0.014981	0.5
L	128	0.5235	0.0000	0.5494	1.0000	0.0000	0.0000	1.0000	0.033669	0.5
L	129	0.9047	0.3390	0.9444	0.0000	1.0000	0.0000	1.0000	0.034888	0.5
L	130	0.8535	0.2349	0.8535	0.0000	0.0000	1.0000	1.0000	0.010161	0.5
L	131	0.8890	0.1483	0.7578	0.4879	0.4954	0.0168	1.0000	0.006345	1
L	132	0.8488	0.0000	0.6177	1.0000	0.0000	0.0000	1.0000	0.003507	0.5
L	133	1.0000	0.3796	1.0000	1.0000	0.0000	0.0000	1.0000	0.03631	0.1
L	134	0.7678	0.1433	0.7678	1.0000	0.0000	0.0000	1.0000	0.01058	1
L	135	0.6830	0.0712	0.6830	0.0000	1.0000	0.0000	1.0000	0.00080	0.5
L	136	0.8825	0.0706	0.6889	1.0000	0.0000	0.0000	1.0000	0.00703	1
L	137	0.6186	0.0000	0.6186	0.0000	0.0000	1.0000	1.0000	0.001323	0.25
L	138	1.0000	0.0000	0.6109	1.0000	0.0000	0.0000	1.0000	0.022068	0.3

L	139	1.0000	0.1558	0.8848	0.0000	1.0000	0.0000	1.0000	0.00874	0.5
L	140	1.0000	0.2343	1.0000	0.5000	0.5000	0.0000	1.0000	0.012799	0.1
L	141	0.7600	0.0000	0.7600	1.0000	0.0000	0.0000	1.0000	0.006012	0.25
L	142	1.0000	0.0000	0.7366	0.5000	0.5000	0.0000	1.0000	0.005993	0.3
L	143	0.9229	0.0804	0.8775	0.0000	1.0000	0.0000	1.0000	0.000544	1
L	144	0.8609	0.0000	0.8609	1.0000	0.0000	0.0000	1.0000	0.006281	0.25
L	145	1.0000	0.0000	1.0000	0.0000	0.0000	1.0000	1.0000	0.058382	0.075
L	146	0.8982	0.0000	0.1374	0.0000	1.0000	0.0000	0.7306	0.034549	0.5
L	147	1.0000	0.1055	0.1055	0.0000	1.0000	0.0000	0.1519	0.0326	0.5
L	148	1.0000	0.6233	0.7554	0.0000	1.0000	0.0000	0.3005	0.02886	0.5
S	1	1.0000	1.0000	1.0000	0.0000	0.0000	1.0000	1.0000	0.036567	0.0583
S	2	0.0000	0.0000	0.0000	0.0000	0.0000	1.0000	1.0000	0.022625	0.0583
S	3	0.6166	0.6166	0.6166	1.0000	0.0000	0.0000	1.0000	0.03383	0.166667
S	4	0.3741	0.3741	0.3741	0.4650	0.4504	0.0846	1.0000	0.025619	0.166667
S	5	0.8141	0.7208	0.8143	0.4756	0.4632	0.0611	1.0000	0.001454	0.5
S	6	0.2647	0.1913	0.1905	0.0000	1.0000	0.0000	1.0000	0.020908	0.5
S	7	0.6205	0.5014	0.6205	1.0000	0.0000	0.0000	1.0000	0.026266	0.5
S	8	1.0000	0.8831	0.8513	0.0000	1.0000	0.0000	0.5000	0.022971	0.5
S	9	0.1189	0.0000	0.1531	0.4912	0.4873	0.0215	1.0000	0.026512	0.25
S	10	0.4987	0.3894	0.3894	1.0000	0.0000	0.0000	1.0000	0.014267	0.5
S	11	0.3844	0.2370	0.3844	0.5798	0.0000	0.4202	1.0000	0.003734	0.5
S	12	0.7680	0.6190	0.6190	0.5964	0.0000	0.4036	1.0000	0.002986	0.5
S	13	1.0000	0.8540	0.8862	0.0000	1.0000	0.0000	0.5000	0.024701	0.5
S	14	1.0000	0.7662	1.0000	0.5000	0.5000	0.0000	1.0000	0.023374	0.2
S	15	0.8568	0.6572	0.7453	1.0000	0.0000	0.0000	1.0000	0.050424	1
S	16	0.2172	0.0000	0.2172	1.0000	0.0000	0.0000	1.0000	0.04403	0.25
S	17	0.2643	0.0000	0.0000	0.5234	0.0000	0.4766	1.0000	0.014926	0.2
S	18	0.6231	0.3866	0.6231	1.0000	0.0000	0.0000	1.0000	0.003165	0.5
S	19	0.6182	0.3811	0.5003	0.7047	0.0000	0.2953	1.0000	0.007213	1
S	20	1.0000	0.7605	0.7365	0.5000	0.5000	0.0000	0.5000	0.021984	0.5

S	21	0.2435	0.0000	0.1523	0.0000	1.0000	0.0000	1.0000	0.033685	0.5
S	22	0.4169	0.1881	0.2557	1.0000	0.0000	0.0000	1.0000	0.014343	1
S	23	0.5861	0.3690	0.3760	1.0000	0.0000	0.0000	1.0000	0.032031	0.5
S	24	1.0000	0.7306	0.7680	0.0000	1.0000	0.0000	0.5000	0.027225	1
S	25	0.8124	0.5037	0.8124	1.0000	0.0000	0.0000	1.0000	0.00349	0.5
S	26	0.5026	0.1938	0.4286	1.0000	0.0000	0.0000	1.0000	0.011372	1
S	27	0.8013	0.4898	0.5655	1.0000	0.0000	0.0000	1.0000	0.00959	1
S	28	0.5014	0.1870	0.1870	0.7210	0.0000	0.2790	1.0000	0.008939	0.5
S	29	0.6145	0.2630	0.6152	1.0000	0.0000	0.0000	1.0000	0.001743	0.5
S	30	0.7306	0.3740	0.3740	1.0000	0.0000	0.0000	1.0000	0.008081	0.5
S	31	1.0000	0.6183	0.7658	1.0000	0.0000	0.0000	1.0000	0.008822	0.5
S	32	0.3860	0.0000	0.2374	1.0000	0.0000	0.0000	1.0000	0.014928	0.5
S	33	1.0000	0.6053	1.0000	1.0000	0.0000	0.0000	1.0000	0.032032	0.2
S	34	1.0000	0.6252	0.8816	1.0000	0.0000	0.0000	1.0000	0.004522	0.5
S	35	0.3868	0.0000	0.3842	0.0000	0.0000	1.0000	1.0000	0.037418	0.25
S	36	0.7760	0.3842	0.6240	1.0000	0.0000	0.0000	1.0000	0.008097	1
S	37	0.6158	0.2291	0.3754	0.7298	0.0000	0.2702	1.0000	0.00494	1
S	38	1.0000	0.6149	0.6149	0.0000	0.0000	1.0000	1.0000	0.005805	0.25
S	39	0.3816	0.0000	0.1173	1.0000	0.0000	0.0000	1.0000	0.005259	0.5
S	40	0.3985	0.0000	0.0000	1.0000	0.0000	0.0000	1.0000	0.042503	0.2
S	41	0.5722	0.1166	0.4272	1.0000	0.0000	0.0000	1.0000	0.011388	1
S	42	0.6108	0.1505	0.6304	0.4102	0.3799	0.2099	1.0000	0.019232	0.5
S	43	0.8482	0.3770	0.7600	1.0000	0.0000	0.0000	1.0000	0.034987	1
S	44	0.8340	0.3808	0.5027	0.0000	1.0000	0.0000	1.0000	0.005585	1
S	45	0.6107	0.1451	0.2259	1.0000	0.0000	0.0000	1.0000	0.033832	1
S	46	0.8533	0.3739	0.3737	1.0000	0.0000	0.0000	1.0000	0.026036	0.5
S	47	1.0000	0.5011	1.0000	1.0000	0.0000	0.0000	1.0000	0.032064	0.2
S	48	1.0000	0.4904	0.7702	0.0000	1.0000	0.0000	0.5000	0.027896	0.5
S	49	0.5002	0.0000	0.2255	0.5000	0.5000	0.0000	1.0000	0.017368	0.5
S	50	0.5005	0.0000	0.0000	1.0000	0.0000	0.0000	1.0000	0.016273	0.2

S	51	0.7691	0.2433	0.6271	0.0000	1.0000	0.0000	1.0000	0.035515	1
S	52	0.7663	0.2447	0.5057	0.0000	1.0000	0.0000	1.0000	0.002018	1
S	53	0.7612	0.2341	0.3728	0.0000	1.0000	0.0000	1.0000	0.030168	1
S	54	0.8120	0.2648	0.8120	1.0000	0.0000	0.0000	1.0000	0.009134	0.5
S	55	0.9970	0.4906	0.8165	0.0000	1.0000	0.0000	0.5000	0.03414	1
S	56	0.7330	0.1846	0.1846	1.0000	0.0000	0.0000	1.0000	0.005164	0.5
S	57	0.7546	0.1396	0.6168	0.0000	1.0000	0.0000	1.0000	0.004572	1
S	58	1.0000	0.3870	1.0000	1.0000	0.0000	0.0000	1.0000	0.038332	0.2
S	59	0.6333	0.0000	0.6333	0.0000	0.0000	1.0000	1.0000	0.034813	0.25
S	60	1.0000	0.3851	0.8478	1.0000	0.0000	0.0000	1.0000	0.018075	0.5
S	61	0.6188	0.0000	0.3400	0.0000	0.0000	1.0000	1.0000	0.045286	0.5
S	62	1.0000	0.4207	0.6026	0.0000	0.0000	1.0000	1.0000	0.059987	0.5
S	63	0.6197	0.0000	0.2344	0.0000	1.0000	0.0000	1.0000	0.032525	0.5
S	64	0.6173	0.0000	0.1216	1.0000	0.0000	0.0000	1.0000	0.007151	0.5
S	65	1.0000	0.3509	0.3509	0.0000	0.0000	1.0000	1.0000	0.060805	0.5
S	66	0.6105	0.0000	0.0000	1.0000	0.0000	0.0000	1.0000	0.038066	0.2
S	67	0.8613	0.2332	0.3677	1.0000	0.0000	0.0000	1.0000	0.008294	1
S	68	0.8089	0.1129	0.6053	1.0000	0.0000	0.0000	1.0000	0.032505	1
S	69	0.8937	0.2312	0.6278	0.0000	1.0000	0.0000	1.0000	0.013581	1
S	70	0.7689	0.1210	0.3845	0.0000	1.0000	0.0000	0.5000	0.024404	1
S	71	0.8873	0.1955	0.8104	1.0000	0.0000	0.0000	1.0000	0.015815	1
S	72	0.8095	0.1150	0.4268	0.0000	1.0000	0.0000	0.5000	0.022575	1
S	73	0.8034	0.1165	0.1906	1.0000	0.0000	0.0000	1.0000	0.00778	1
S	74	0.7490	0.0000	0.7546	1.0000	0.0000	0.0000	1.0000	0.029904	0.25
S	75	1.0000	0.2606	0.2354	0.0000	1.0000	0.0000	1.0000	0.030194	0.5
S	76	1.0000	0.2705	1.0000	0.5119	0.0000	0.4881	1.0000	0.011688	0.2
S	77	0.7715	0.0000	0.6273	1.0000	0.0000	0.0000	1.0000	0.016214	0.5
S	78	1.0000	0.2347	0.7656	0.6249	0.0000	0.3751	1.0000	0.013864	0.5
S	79	0.7581	0.0000	0.5090	0.5000	0.5000	0.0000	1.0000	0.012018	0.5
S	80	1.0000	0.2320	0.6182	0.0000	1.0000	0.0000	1.0000	0.029963	0.5

S	81	0.7676	0.0000	0.3865	0.0000	1.0000	0.0000	1.0000	0.027702	0.5
S	82	1.0000	0.2305	0.4993	0.5000	0.5000	0.0000	1.0000	0.023557	0.5
S	83	0.7656	0.0000	0.2356	1.0000	0.0000	0.0000	1.0000	0.015771	0.5
S	84	1.0000	0.2331	0.3789	1.0000	0.0000	0.0000	1.0000	0.021104	0.5
S	85	0.7397	0.0000	0.0000	0.5120	0.0000	0.4880	1.0000	0.005249	0.2
S	86	0.8469	0.0000	0.8886	0.4804	0.4721	0.0475	1.0000	0.030208	0.25
S	87	0.8620	0.0000	0.7748	0.0000	1.0000	0.0000	1.0000	0.034707	0.5
S	88	1.0000	0.1152	0.6192	1.0000	0.0000	0.0000	1.0000	0.013057	0.5
S	89	0.8506	0.0000	0.3863	1.0000	0.0000	0.0000	1.0000	0.025576	0.5
S	90	0.8586	0.0000	0.1414	1.0000	0.0000	0.0000	1.0000	0.023994	0.5
S	91	0.8820	0.0000	0.6190	1.0000	0.0000	0.0000	1.0000	0.007789	0.5
S	92	1.0000	0.1186	0.3797	1.0000	0.0000	0.0000	1.0000	0.008419	1
S	93	1.0000	0.0000	1.0000	0.0000	0.0000	1.0000	1.0000	0.029466	0.025
S	94	1.0000	0.0000	0.7665	0.5003	0.0000	0.4997	1.0000	0.023321	0.1
S	95	1.0000	0.0000	0.6100	1.0000	0.0000	0.0000	1.0000	0.028762	0.1
S	96	1.0000	0.0000	0.5002	1.0000	0.0000	0.0000	1.0000	0.026407	0.1
S	97	1.0000	0.0000	0.3870	1.0000	0.0000	0.0000	1.0000	0.033026	0.1
S	98	1.0000	0.0000	0.2343	0.5036	0.0000	0.4964	1.0000	0.017773	0.1
S	99	1.0000	0.0000	0.0000	0.0000	0.0000	1.0000	1.0000	0.029022	0.025
S	100	1.0000	0.1349	0.8564	1.0000	0.0000	0.0000	1.0000	0.011128	0.5
S	101	1.0000	0.0966	0.9330	0.0000	1.0000	0.0000	0.7037	0.02959	0.5
S	102	1.0000	0.4646	0.5343	1.0000	0.0000	0.0000	0.9609	0.01795	0.5
S	103	0.5291	0.0000	0.4369	1.0000	0.0000	0.0000	0.9353	0.036378	0.5
S	104	0.9202	0.0000	0.0974	0.0000	1.0000	0.0000	0.3474	0.028711	0.5
